# Granulopoietic Dysregulation in a Patient-Tailored Mouse Model of Barth Syndrome

**DOI:** 10.1007/s12015-025-10945-1

**Published:** 2025-08-05

**Authors:** Elizabeth A. Sierra Potchanant, Maegan L. Capitano, Donna M. Edwards, Baskar Ramdas, Scott Cooper, James Ropa, S. Louise Pay, Aditya Sheth, Paige L. Snider, Hilary J. Vernon, Ngoc-Tung Tran, Reuben Kapur, Simon J. Conway

**Affiliations:** 1 Department of Pediatrics, Herman B. Wells Center for Pediatric Research, Indiana University School of Medicine, 1044 West Walnut Street, Indianapolis, IN 46202 USA; 2 Department of Microbiology & Immunology, Indiana University School of Medicine, 635 Barnhill Drive, Indianapolis, IN 46202 USA; 3 Department of Medical & Molecular Genetics, Indiana University School of Medicine, 410 West 10th Street, Indianapolis, IN 46202 USA; 4 Stark Neurosciences Research Institute, Indiana University School of Medicine, 320 West 15th Street, Indianapolis, IN 46202 USA; 5 Department of Genetic Medicine, Johns Hopkins University School of Medicine, 733 North Broadway, Baltimore, MD 21205 USA

**Keywords:** Patient-tailored mouse model, Tafazzin, Barth syndrome, Neutropenia, Hematopoietic stem cells

## Abstract

Barth syndrome (BTHS) is an X-linked recessive disorder characterized by cardiomyopathy, skeletal muscle myopathy and fatigue, growth restriction, and neutropenia. Neutropenia increases the risk of life-threatening bacterial infections, a major cause of death in individuals with BTHS. Currently, there is no curative treatment for BTHS or associated neutropenia. The development of therapeutic strategies to correct BTHS-associated neutropenia has been hindered by a limited understanding of the underlying molecular mechanisms involved. BTHS is caused by a mutation in the *Tafazzin* gene encoding a transacylase required for the maturation of cardiolipin, an inner mitochondrial membrane phospholipid crucial for mitochondrial structure and function. We introduced a BTHS patient’s point mutation (*TAZ*^*D75H*^) into the mouse Tafazzin enzyme’s critical acyltransferase site using *CRISPR/Cas9*-mediated genome editing, resulting in a patient-tailored point mutant knock-in BTHS model (*Taz*^*D75H*^) that expresses a stable mutant Taz^D75H^ protein lacking transacylase activity. *Taz*^*D75H *^mice were then used to investigate how loss of Tafazzin enzymatic activity impacts hematopoiesis. Male *Taz*^*D75H*^ mice exhibited impaired granulopoiesis and neutropenia secondary to impaired function of hematopoietic progenitors. Furthermore, they demonstrated age-dependent neutrophil maturation impairment reflecting the variable neutropenia observed in BTHS patients. Additionally, male *Taz*^*D75H*^ mice exhibit chronic lymphopenia that persists post *Taz*^*D75H*^ bone marrow transplantation. Mechanistically, the *TAZ*^*D75H*^ point mutation caused hematopoietic cell mitochondrial dysfunction in patient-derived immortalized *TAZ*^*D75H*^ lymphoblasts, increasing reactive oxygen species production and mitochondrial membrane depolarization. Likewise, Cyclosporine A treatment rescued these mitochondrial phenotypes in vitro, confirming *TAZ*^*D75H*^ mitochondrial dysfunction. Overall, our findings demonstrate that mitochondrial dysfunction secondary to TAFAZZIN loss of enzymatic function underlies BTHS-associated neutropenia and lymphopenia.

**Supplementary Information:**

The online version contains supplementary material available at 10.1007/s12015-025-10945-1.

## Introduction

Barth syndrome (BTHS; OMIM# 302060) is a rare, life-threatening Xq28-linked mitochondrial disorder caused by mutations in the *Tafazzin* gene. BTHS primarily affects males and is characterized by cardiomyopathy, neutropenia, skeletal myopathy, and failure to thrive, typically presenting in infancy or early childhood [1–5]. BTHS is the most common X-linked mitochondrial disorder, and as with most mitochondriopathies, there is no cure [5, 6].

*TAFAZZIN* encodes an enzymatic transacylase required for the formation of mature cardiolipin (CL), a prominent inner mitochondrial membrane (IMM) phospholipid [3–7]. CL is vital for maintaining proper mitochondrial oxidative phosphorylation (OxPhos) in skeletal muscle, including electron transport chain activity and ATP production, as well as mitochondrial morphology and mitophagy [3–9]. BTHS patient tissues and cells exhibit reduced CL levels, altered mitochondrial structure, and decreased OxPhos functional capacity [4, 7, 9]. More than 200 different *TAFAZZIN* mutations cause BTHS, with variable outcomes; most result in mutant TAFAZZIN proteins [10–13]. Moreover, the TAFAZZIN protein performs nonenzymatic functions; it senses IMM curvature, is essential for glycerolipid acyltransferase catalysis, and binds various adenine nucleotide metabolism and protein complexes at its hydrophilic domain [14–17]. Consequently, the spectrum and severity of BTHS symptoms can vary, genotype‒phenotype correlations are incompletely understood, and the natural course of disease progression throughout the patient’s lifetime remains unclear [3, 5–18].

Among the most serious complications of BTHS is neutropenia, a pathological disease resulting from lower-than-normal circulating neutrophil levels, increasing infection risk [19–21]. Neutropenia can present before birth in BTHS patients [22] and is often the initial finding [23, 24]; furthermore, patients often succumb to bacterial infections secondary to repeated life-threatening infections [5, 20, 21]. However, the mechanisms driving neutropenia in BTHS are not well elucidated [21, 23], and its presentation is variable. Some patients do not exhibit neutropenia [5, 25], whereas others demonstrate intermittent and unpredictable, chronic and severe, or cyclical presentations [23, 24]; as well as compensatory monocytosis help to compensate for neutrophil deficiency [20, 23]. Thus, there is an urgent need to characterize neutropenia in patient-derived models to enable the development of targeted therapies for BTHS and other neutropenia conditions.

Neutrophils are the most prevalent circulatory immune cells involved in the innate immune response to infection and tissue remodeling [25, 26]. These cells are short-lived [27], necessitating continual replenishment from bone marrow hematopoietic stem cells (HSCs). BTHS-derived lymphoblasts exhibit reduced CL levels and abnormal mitochondrial structures [28]. Nevertheless, it remains unclear whether BTHS neutropenia arises from defective granulopoiesis, increased neutrophil apoptosis, accelerated clearance, or functional defects.

In this study, we aimed to address this knowledge gap in a patient-tailored mouse model and patient-derived lymphoblasts. We examined granulopoiesis in a mouse model expressing a novel patient-derived *TAFAZZIN* variant (*D75H* substitution within the H(X)_4_D acyltransferase motif; *Taz*^*D75H*^ [13]) generated by *CRISPR/Cas9* genetic modification and in immortalized *TAZ*^*D75H*^ lymphoblasts from this BTHS patient. Our study provides a patient-relevant platform for elucidating the mechanisms of neutrophil dysregulation in BTHS and identifying potential therapeutic targets.

## Methods

### Animal Models

*Tafazzin* (MGI:109626) point mutant knock-in mice (herein termed *Taz*^*D75H*^) with a pathogenic mutation (D75H: G > C substitution within Asp223 in coding exon 2) were generated via *CRISPR/Cas9* gene editing, maintained on a C57BL/6J background and bred/genotyped as previously described [13]. BoyJ male mice used for transplantation were obtained from the onsite Indiana University Simon Comprehensive Cancer Center (IUSCCC) In Vivo Therapeutics Core. All the mice were housed in a specific pathogen-free facility in the Animal Resource Center at IU School of Medicine, and the Institutional Animal Care and Use Committee of IU School of Medicine approved all the experimental procedures.

### Cytospin Preparation and Wright‒Giemsa Staining

Venous blood was drawn from the lateral tail veins of 5-day-old, 4-week-old, and 8- to 10-month-old *wt* male (*♂*) and *Taz*^*D75H*^♂ littermates and collected in EDTA-coated tubes. Complete blood counts (CBCs) were obtained via a Hemavet 950FS (Drew Scientific), as described [13]. Primary bone marrow was flushed from 4-week-old *wt♂* and *Taz*^*D75H*^♂ littermate femurs and tibias and then processed to create a single-cell suspension suitable for cytospinning. Blood and cells were spun onto glass slides (Cytospin 4 Cytocentrifuge, Thermo Scientific), dried for 20 min, fixed in methanol, and stained with either Giemsa (Sigma) or Wright‒Giemsa (Siemens). Images were acquired with an Axioscope2 (Zeiss) instrument equipped with a 100× oil immersion objective, and the image brightness was adjusted with Photoshop (Adobe).

### Histology

10 μm frozen *wt*♂ and *Taz*^*D75H*^♂ adult spleens sections (by Leica CM3000 Cryostat) were collected on Superfrost plus slides (VWR) and air dried for 30–90 min (*n* = 3 spleens/genotype). Following brief 4% paraformaldehyde fixation and PBS washing, sections were probed with anti-neutrophil (Ly-6G and Ly-6 C) antibody (1:400, Abcam, clone NIMP-R14) and signal detected using the ABC kit and a rat-specific secondary antibody (Vectorstain). DAB/hydrogen peroxide was used to visualize immunosignals. Antibody diluent (Vectorstain), without primary antibodies, was used for negative control. For each assay, serial sections were examined, and a specific signal was only noted when present in at least three consecutive serial sections.

### Flow Cytometry Analysis

Bone marrow-derived HSCs and progenitor (HPC) cell phenotypes were identified via flow cytometry in single-cell suspensions from four-week-old ♂ mice. The following phenotyping markers were used: APC, FITC or Pacific Blue™ mouse lineage cocktail (CD3, Gr-1, CD11b, CD45R, and Ter119; APC cocktail from BD Bioscience; FITC and Pacific Blue™ cocktail from BioLegend), PE-CF594-anti-Ly6A/E (a.k.a. Sca-1; clone D7; BD Biosciences), APC or APC-H7-anti-CD117 (a.k.a. c-Kit; clone 2B8; BD Biosciences), APC- or PE-anti-CD135 (a.k.a. Flt3; clone A2F10.1; BD Biosciences), PE- or BV421-anti-CD34 (clone RAM34; BD Biosciences), PerCP-Cy™5.5-anti-CD16/CD32 (a.k.a. FcγR; clone 2.4G2; BD Biosciences), and BV421-anti-CD127 (a.k.a. IL-7R; clone SB/199; BD Biosciences). The HSC and HPC populations were defined as LK- or myeloid progenitor-enriched cells: Lin-Sca-1-c-Kit+, LSK cells: Lin- Sca-+ c-Kit+, long-term (LT)-HSCs: LSK Flt3- CD34-, short-term (ST)-HSCs: LSK Flt3- CD34+, multipotent progenitors (MPPs): LSK Flt3+ CD34+, common myeloid progenitors (CMPs): LK FcγRlo CD34+, granulocyte- monocyte progenitors (GMPs): LK FcγRhi CD34+, megakaryocyte-erythrocyte progenitors (MEPs): LK FcγR- CD34-, and common lymphoid progenitors (CLPs): Lin- Sca-1lo c-Kitlo Flt3+ IL-7R+. Neutrophils were defined as Ly-6G+ (APC anti-Ly-6G; clone 1A8; BioLegend) and CD11b+ (FITC anti-CD11b; clone M1/70; BioLegend). Experiments were performed on a modified FACSLSR II cytometer (BD Biosciences) and analyzed with FlowJo version 7.6.3 software (TreeStar).

MitoTracker™ Green FM (Invitrogen) staining was performed according to the manufacturer’s instructions and as described previously [29–31]. JC-1 staining, utilizing a MitoProbe™ JC-1 Assay Kit (Invitrogen), was performed according to the manufacturer’s instructions and as described previously [29]. To detect mitochondrial superoxide, we incubated ♂ lymphoblasts in medium containing MitoSOX reagent (Invitrogen) at 37 °C for 20 min, washed twice, and then analyzed via flow cytometry. Positive control cells were incubated in medium containing 9 µM H_2_O_2_ for 50 min prior to the addition of MitoSOX.

### Hematopoietic Progenitor Cell Colony and Tritiated Thymidine Kill Assays

Hematopoietic clonogenic progenitors from 4-week-old *wt♂* and *Taz*^*D75H*^♂ bone marrow cells flushed from femurs were grown in 1% methylcellulose culture medium with 0.1 mM hemin (Sigma-Aldrich), 30% FBS, 1 U/mL recombinant human erythropoietin (rhEPO; Amgen), 50 ng/mL recombinant mouse stem cell factor (rmSCF; R&D Systems), and 5% vol/vol pokeweed mitogen mouse spleen cell conditioned medium at 5 × 10^4^ cells/mL per plate (3 plates per mouse). Colonies were scored after 6 days of incubation in 5% CO_2_ and low (5%) O_2_ in a humidified chamber. Granulocyte‒macrophage colony-forming units (CFU‒GM), erythrocyte burst-forming units (BFU‒E), and granulocyte, erythrocyte, macrophage, and megakaryocyte colony-forming units (CFU‒GEMM) were distinguished by examining colony morphology. The total number of colonies per femur was calculated as previously described [30, 31]. For high-specific activity tritiated thymidine kill assays, bone marrow cells were treated with 50 µCi high-specific activity [^3^H]Tdr (20 Ci/mmol; DuPont NEN) at room temperature (20–22 °C) for 40 min and then washed in PBS before plating for HPC colony assays (as previously described [31, 32]).

### Bone Marrow Transplantation and ROS Assessment

Bone marrow were harvested from either 4-week-old or 6-month-old *wt♂* and *Taz*^*D75H*^♂ mice, and low-density mononuclear cells (LDMNCs) were isolated as previously described [29–33]. Recipient male BoyJ mice (6‒8 weeks old) from the onsite IUSCCC In Vivo Therapeutics Core were subjected to whole-body irradiation with 1,100 cGy (*n* = 10/genotype) and transplanted intravenously with 2 × 10^6^ donor bone marrow LDMNCs after 24 h. For in vivo hematopoietic assessment, CBC analysis was performed 1 to 10 months post-transplantation. For reactive oxygen species (ROS) examination, 8-weeks post transplantation, marrow was harvested and ROS levels assessed using the CellROX Deep Red Reagent, as per the manufacturer’s instructions (Thermo Fisher Scientific). Briefly, single-cell suspensions were prepared from freshly isolated adult marrow. After red blood cell lysis and washing, cells were resuspended in pre-warmed Roswell Park Memorial Institute (RPMI) containing 10% FBS. CellROX Deep Red was added at a final concentration of 3 µM, and cells were incubated at 37 °C for 30 min in the dark. Following incubation, cells were washed twice with ice-cold HBSS/FBS buffer and surface immunostaining for hematopoietic markers was performed on ice using fluorophore-conjugated antibodies (Lineage cocktail BV421, Sca-1-BV711, KIT-BV785, BioLegend) and cells were washed with HBSS with + 2% FBS to remove unbound antibodies, and immediately analyzed by flow cytometry without fixation.

### BTHS Patient Lymphoblast Line and BTHS Patient Blood Data

*TAZ*^*D75H*^ patient lymphocytes were isolated from fresh blood samples and subjected to Epstein–Barr virus transformation as previously described [34]. Control EBV-immortalized human lymphoblasts (NCI-BL1672) were obtained from the American Type Culture Collection. Both cell types were cultured in RPMI medium supplemented with 10% FBS, 1% sodium pyruvate, and 1% penicillin–streptomycin. Lymphocyte collection and transformation were approved by the Johns Hopkins University Institutional Review Board (NA_00098987: ‘Investigation into clinical, metabolic and molecular factors in Barth syndrome’; PI H.J. Vernon). For the collection of BTHS data, the study was longitudinal and observational in nature. This study was approved by the Johns Hopkins University IRB protocol (NA_0008316: ‘Clinical Studies in Barth Syndrome’; PI H.J. Vernon). All procedures were conducted per the ethical standards of the responsible committee on human experimentation (institutional and national) and the tenets of the Declaration of Helsinki of 1975, as revised in 2013. Informed written consent was obtained from the parents or legal guardians of all participants in the study.

### Transmission Electron Microscopy

For electron microscopy (EM), patient *TAZ*^*D75H*^ lymphoblasts were placed on coverslips and fixed in 2% glutaraldehyde fixative in 0.1 M cacodylate buffer (pH 7.2) overnight, processed, and imaged on a Phillips400 microscope at the IU EM Core as previously described [13]. Examiners were blinded to the genotype, and the data were analyzed via ImageJ (version 1.54j).

### Detection of Oxidative Phosphorylation Complexes

Mitochondria were isolated from human lymphoblasts using the Mitochondria Isolation Kit for Cultured Cells (Abcam, ab110171) as per manufacturer’s instructions. Mitochondria were lysed using M-PER buffer Mammalian Protein Extraction Reagent (Thermo Fisher Scientific) containing protease and phosphatase inhibitors. The lysates were denatured for 15 min at 37 °C and proteins were resolved using a 10–20% gradient gel (Novex™, Thermo Fisher Scientific). Protein was transferred to PVDF using high pH CAPS transfer buffer, and blots were probed with Total OXPHOS Rodent WB Antibody Cocktail (1:250, Abcam) for intact electron transport complexes as per the manufacturer’s instructions. VDAC1 (1:2000, Abcam) was used as loading control.

### Cyclosporine A Treatment

Cyclosporine A (Cayman Chemical) was dissolved in DMSO and added to the lymphoblast cultures at a final concentration of 10 µM. An equal volume of pure DMSO was added to control cells. The total exposure duration was 90 min at 37 °C.

### Statistical Analyses

Statistical analyses were performed using GraphPad Prism 7 (GraphPad Software). One-way ANOVA with post hoc Tukey’s multiple comparison test or Student’s *t-test* were used to determine significant differences between groups. A *p* value below 0.05 was considered to indicate significance. The ROUT test (Robust regression and Outlier Removal Test) on GraphPad was used to determine any outliers. The data in the figures are presented as the means ± SEMs.

## Results

### Impaired Granulopoiesis and Neutropenia in Taz^D75H^ Male Mice

We identified a novel *TAFAZZIN* variant causing a *D75H* substitution in the critical acyltransferase site of the TAFAZZIN enzyme in a 1.5-year-old male BTHS patient exhibiting growth deficiency, hypotonia, gross motor delay, and noncompaction cardiomyopathy. Significantly, additional BTHS mutations are present around residue *D75 (D74E*,* D75N*,* D75R*,* D76R*,* D76L*) at the border of the H(X)_4_D acyltransferase domain, and ~ 47% of residues in proximity to *D75* carry pathogenic mutations [10]. Importantly, this *TAZ*^*D75H*^ patient initially presented with recurrent fever, monocytosis and neutropenia and requires continual G-CSF therapy [35] to stimulate his bone marrow to induce granulopoiesis and neutrophil release to help prevent infections. Thus, we examined baseline hematopoiesis in a *CRISPR/Cas9* knock-in mutant mouse model harboring this *D75H* point mutation (referred to hereafter as *Taz*^*D75H*^) in the critical TAZ acyltransferase domain required to remodel immature monolysocardiolipin (MLCL) to mature (18:2)_4_ cardiolipin (CL) [13].

Male *Taz*^*D75H*^ mice exhibited BTHS phenotypes and an elevated adult blood MLCL: CL ratio (diagnostic feature of BTHS [7]). Furthermore, only ~ 50% of *Taz*^*D75H*^♂ mice survived perinatally (the other ~ 50% die *in utero* or at birth likely due to cardiovascular defects [36, 37]), whilst the remaining *Taz*^*D75H*^♂ all die prematurely by ~ 11 months of age [13]. Thus, we analyzed surviving neonatal, juvenile, and mature adult *Taz*^*D75H*^♂ mice. Peripheral blood smear assessment revealed that both 5-day-old and 4-week-old *Taz*^*D75H*^♂ mice exhibit significantly fewer neutrophils than their *wt*♂ littermates, whereas 8–10-month-old adult *Taz*^*D75H*^♂ and *wt*♂ mice exhibit similar neutrophil levels (Supplemental Fig. 1 A, B). These data suggest that the mild-to-moderate neutropenia mediated by the point mutation in *Taz*^*D75H*^♂ mice is apparent only in young mice and likely present from birth.

**Fig. 1:**
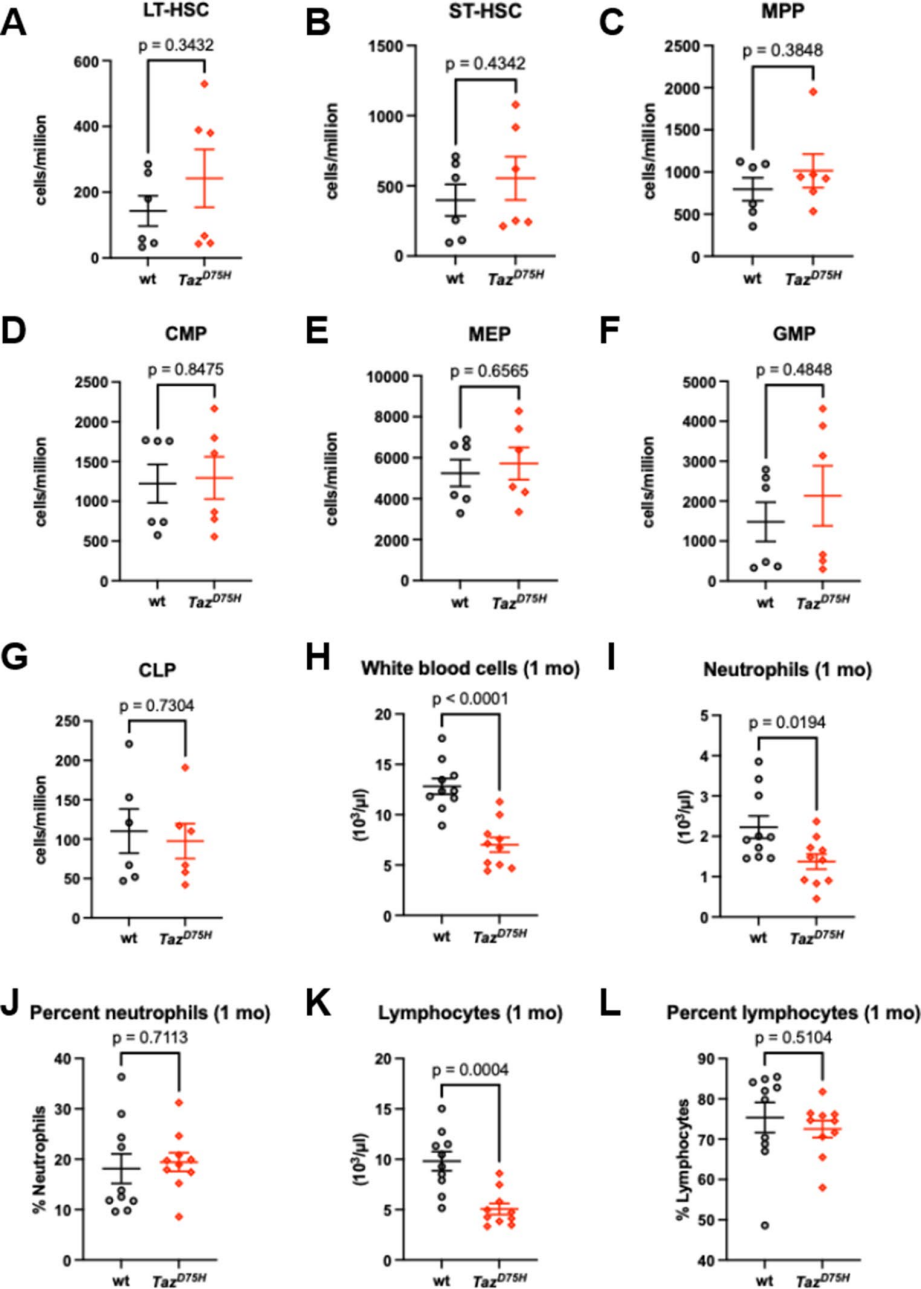
Hematopoiesis is impaired in *Taz*^*D75H*^♂ male mice. (**A**-**G**) Quantitative flow cytometry immunophenotyping of *Taz*^*D75H*^♂ and *wt*♂ mouse bone marrow reveals the relative abundance of long- and short-term HSCs (A, B) and lineage-specific myeloid progenitors (C–G). The graphs present pooled data (means ± SEMs) from two independent experiments (*n* = 3/genotype/experiment). *P* values were calculated by *t-test*. (**H**-**L**) CBCs in 3-5-week-old (1 mo) *Taz*^*D75H*^♂ and *wt*♂ mice revealed significant differences in the absolute numbers of total white blood cells, neutrophils, and lymphocytes (H, I, K). Graphs represent means ± SEM (*n* = 10 mice/genotype), and *p* values were calculated by *t-test*

Consistent with prior published data [13], we observed significant variability in neutrophil number reduction in neonatal and juvenile *Taz*^*D75H*^♂ mice, reflecting the variability observed in BTHS [2, 5, 19, 23]. Given these peripheral smear data, we examined cytopsin preparations from 4-week-old mice and revealed that the bone marrow of both *Taz*^*D75H*^♂ and *wt*♂ mice exhibit a comparable spectrum of differentiating neutrophil precursors, including a significant number of immature band and ring neutrophils with some mature segmented neutrophils in both genotypes (Supplemental Fig. 1 C). This finding indicates that immature myeloblast precursors in *Taz*^*D75H*^♂ mice can at least undergo preliminary differentiation. Additionally, we immunophenotypically quantified HSCs and HPCs in the bone marrow of 4-week-old *Taz*^*D75H*^♂ and *wt*♂ mice via flow cytometry (Fig. 1). Compared to *wt*♂ littermate controls, long-term HSCs (LT-HSCs) and short-term HSCs (ST-HSCs) in *Taz*^*D75H*^♂ mice were not significantly different (Fig. 1A, B). Similarly, there were no significant differences in the numbers of MPPs, CMPs, GMPs, MEPs, or CLPs in *Taz*^*D75H*^♂ versus *wt*♂ mouse bone marrow samples (Fig. 1C–G). Interestingly, Complete blood counts (CBC) analyses revealed that 4-week-old *Taz*^*D75H*^♂ mice exhibited a significant reduction in the absolute number of neutrophils (*p* = 0.0194 by *t-test*; *n* = 10 mice/genotype; Fig. 1I). Overall, *Taz*^*D75H*^♂ mice presented decreased absolute white blood cell (WBC) and lymphocyte counts (Fig. 1H, K). Taken together, these data suggest that the *Taz*^*D75H*^ mutation results in neonatal/juvenile dysfunctional maturation at the final neutrophil differentiation step under homeostatic conditions.

### Ex Vivo Hematopoietic Progenitor Cell Colony Analysis

**Fig. 2:**
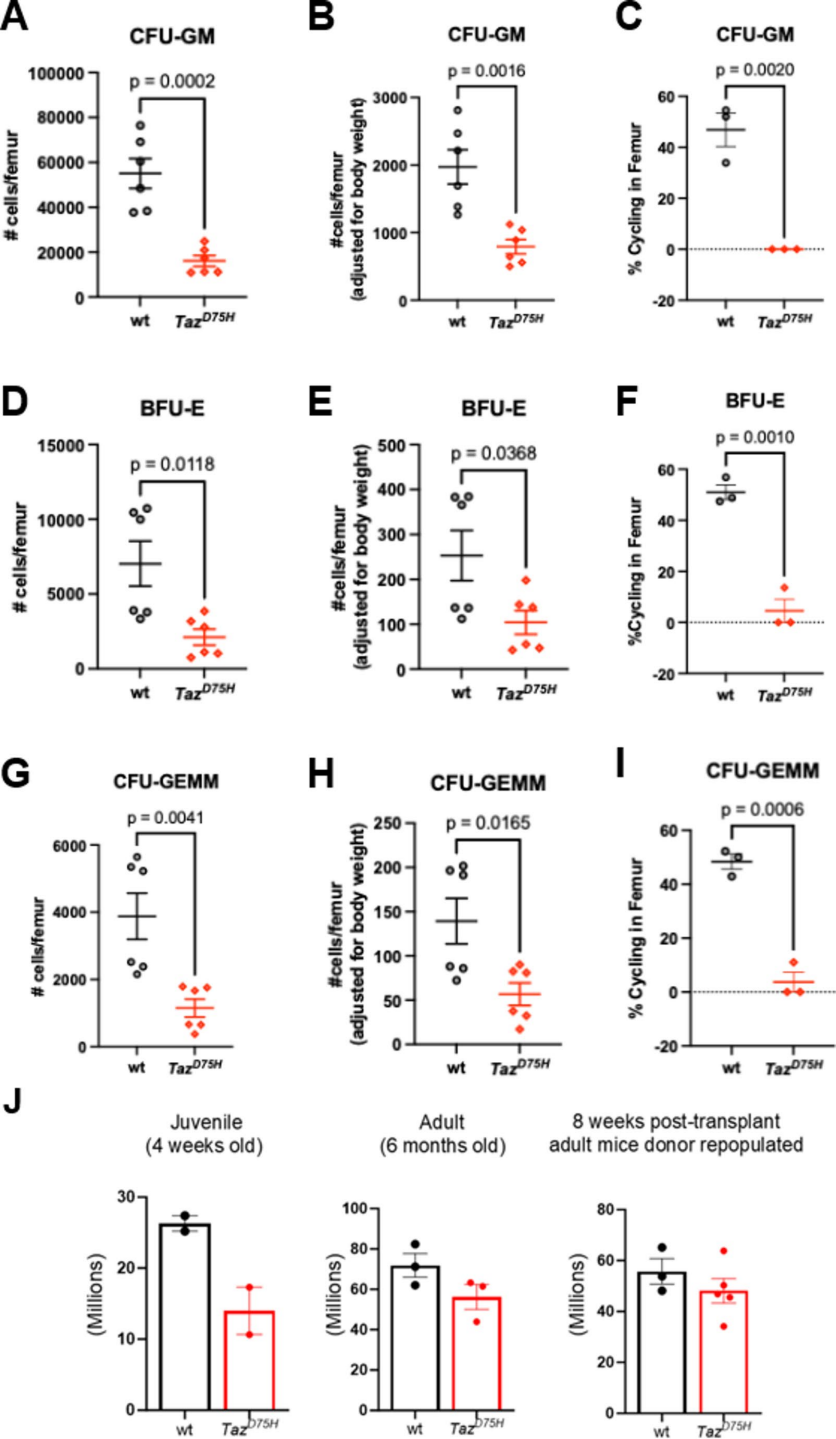
Decreased function of hematopoietic myeloid progenitors in *Taz*^*D75H*^♂ bone marrow. Colony-forming units (CFUs) for granulocyte-macrophage (**A**), burst-forming unit-erythrocyte (**D**), and granulocyte-erythrocyte-macrophage-megakaryocyte (**G**) numbers per femur of *Taz*^*D75H*^♂ and *wt*♂ mice as determined by colony assay. (**B**, **E**, **H**) Body weight-adjusted numbers of bone marrow CFUs. (**C**, **F**, **I**) The percentage of HPCs cycling per femur was determined via a thymidine kill assay. For the CFU data, the graphs represent pooled data (means ± SEMs) from two independent experiments (*n* = 3/genotype/experiment). For the thymidine kill data, the graphs represent data from a single experiment (*n* = 3/genotype). (**J**) Total bone marrow cellularity (total number of bone marrow cells/mouse) of juvenile and adult *Taz*^*D75H*^♂ and *wt*♂ bone marrow, as well as adult *Taz*^*D75H*^♂ and *wt*♂ bone marrow 8 weeks post-transplantation. (*n* = 2–5 mice/genotype)

To evaluate the functionality of hematopoietic myeloid progenitors in *Taz*^*D75H*^♂ mice, we performed ex vivo HPC colony assays, as previously described [32]. Given the observed chronic growth deficiency [13] of *Taz*^*D75H*^♂ mice, the data are shown both as absolute cell number/femur and as adjusted to body weight (Fig. 2). These assays revealed impaired differentiation within all myeloid progenitors examined in the bone marrow of 4-week-old *Taz*^*D75H*^♂ versus *wt*♂ mice. Compared with that in *wt*♂ mice, the number of myeloid progenitor cells capable of forming colonies consisting of 20 or more granulocytes and macrophages (GMPs/CFU-GM) was significantly reduced in the bone marrow of *Taz*^*D75H*^♂ versus *wt*♂ mice (Fig. 2A, B). Similarly, the number of CMPs capable of forming colonies containing erythroid, megakaryocyte, granulocyte, and megakaryocyte (CFU-GEMM) cells was reduced in *Taz*^*D75H*^♂ versus *wt*♂ mice (Fig. 2G, H), as were the number of cells capable of forming colonies consisting predominantly of erythroblasts (BFUs; Fig. 2D, E). The decrease in CFU-GM, BFU-E, and CFU-GEMM numbers in the *Taz*^*D75H*^♂ mouse femurs in the colony formation assays was associated with a decrease in the number of myeloid progenitors in the S-phase at the time of plating, as defined by the high specific activity of the tritiated thymidine kill assay (Fig. 2C, F, I). In order to examine whether mutant bone marrow cellularity itself was perturbed by the *Taz*^*D75H*^ mutation, CBCs revealed 4-week-old *Taz*^*D75H*^♂ bone marrow exhibited a trend toward reduced cellularity, suggesting a transient impairment during early hematopoietic development (Fig. 2J). In contrast, adult *Taz*^*D75H*^♂ displayed normal bone marrow cellularity, indicating that the early deficit is resolved over time. Moreover, bone marrow cellularity in *Taz*^*D75H*^♂ recipients at 8 weeks post-transplant was comparable to *wt*♂ recipients (Fig. 2J), further supporting the notion that any early defects are not sustained, and that the mutant marrow environment can recover or compensate functionally under steady-state and regenerative conditions.

In parallel, we assessed the colony-forming ability of HPC populations from splenocytes isolated from *Taz*^*D75H*^♂ or *wt*♂ littermates. Interestingly, we did not observe decreased colony-forming ability in splenocyte-derived HPCs from *Taz*^*D75H*^ mice (Supplemental Fig. 2). Notably, when adjusted for body weight, CFU-GM colony formation was significantly increased in *Taz*^*D75H*^♂ versus *wt*♂ mice, as was the percentage of CFU-GM in S-phase at the time of plating (Supplemental Fig. 2B, C), suggesting a potential compensatory mechanism for the impaired bone marrow granulopoiesis and subsequent neutropenia observed in juvenile *Taz*^*D75H*^♂ mice. However, the CBC results indicate that this extramedullary hematopoiesis in the spleen does not fully compensate for the loss of function of the myeloid progenitor cells producing neutrophils in the mutant bone marrow. Immunohistochemistry using an anti-neutrophil marker antibody revealed that cellularity and neutrophil numbers were comparable in *Taz*^*D75H*^♂ and *wt*♂ adult spleens (Supplementary Fig. 2 J). Whether this is due to differences in adhesion or migration or perhaps excessive secretion of cytokines responsible for mobilizing HSPCs and/or neutrophils remains to be determined. Taken together, these data suggest an impaired mutant capacity to generate and differentiate bone marrow HPCs into mature neutrophils, which likely underlies the *Taz*^*D75H*^♂ neonatal/juvenile neutropenia phenotype and diminished absolute WBC/lymphocyte counts.

### In Vivo Hematopoiesis Analysis

**Fig. 3:**
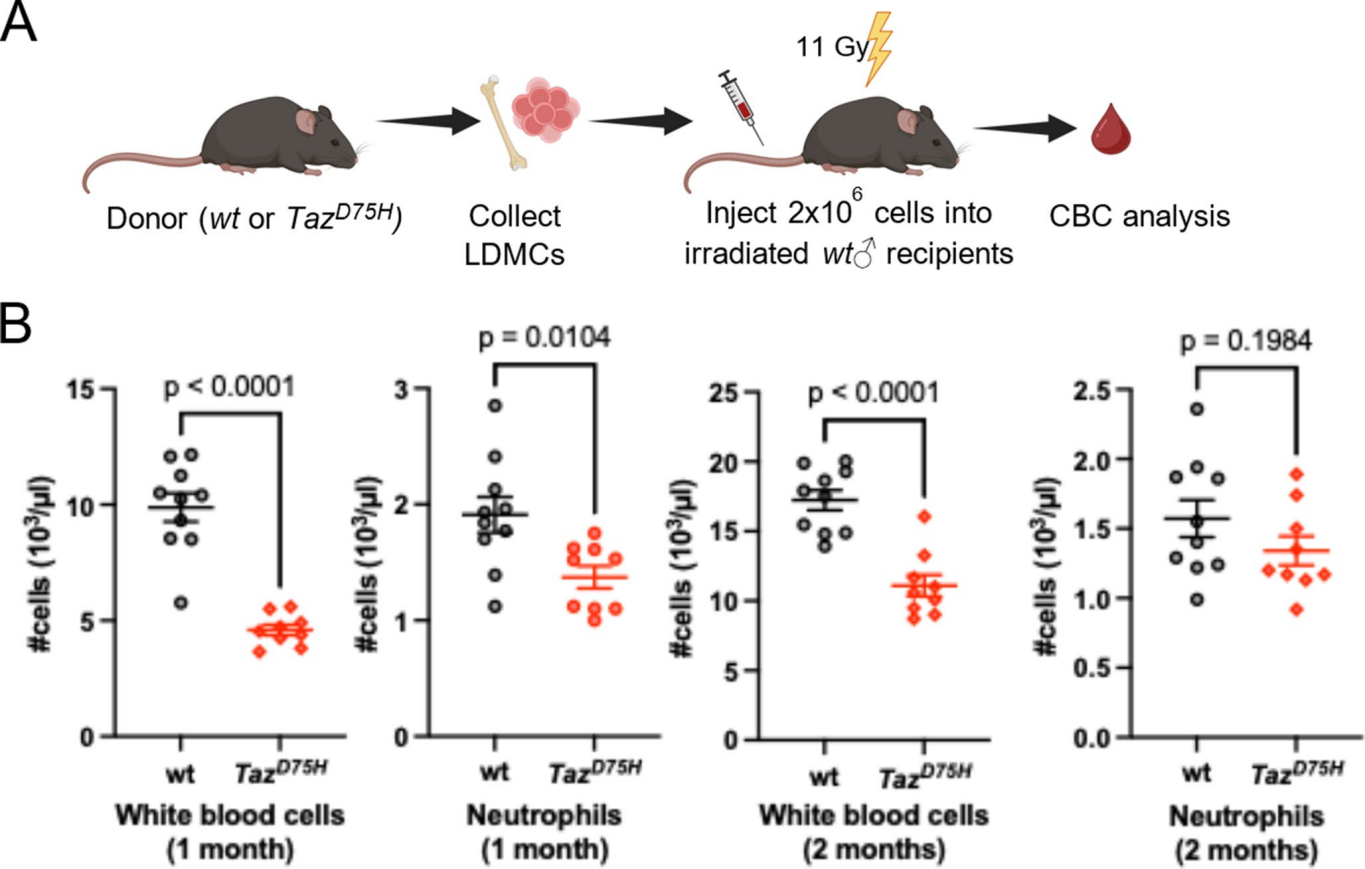
The *Taz*^*D75H*^ mutation induces impaired granulopoiesis in vivo*.* (**A**) Schematic of the bone marrow transplantation experiments. LDMNCs collected from *Taz*^*D75H*^♂ and *wt*♂ mice were transplanted into irradiated *wt*♂ recipients (*n* = 10 recipient mice/genotype). Peripheral blood was collected for CBC analysis at one- and two-months post transplantation. (**B**) Plots showing the quantification of the indicated cell types. *P* values were calculated via unpaired *t-tests*. Results from one *Taz*^*D75H*^♂ recipient mouse were identified as outlier by the ROUT *t-test* and were thus omitted from the statistical analysis

To directly assess the impact of the *Taz*^*D75H*^ mutation on hematopoiesis in vivo, we transplanted low-density mononuclear cells (LDMCs) isolated from the bone marrow of 4-week-old *Taz*^*D75H*^♂ or *wt*♂ mice into lethally irradiated *wt*♂ recipient mice (6–8 weeks of age, Fig. 3A). One month after transplantation, CBC analysis of peripheral blood revealed significant reductions in total WBCs and neutrophils in *Taz*^*D75H*^♂ bone marrow recipients (Fig. 3B). However, 2 months after transplantation, recipients of *Taz*^*D75H*^♂ bone marrow no longer exhibited neutropenia, despite a sustained significant reduction in total WBC count (Fig. 3B and Supplemental Fig. 3). These data suggest that the neutropenia is temporary even through transplantation, confirming a correction in neutrophil production with time. Moreover, CBC analysis of *Taz*^*D75H*^♂ and *wt*♂ mice revealed *Taz*^*D75H*^♂ reduced RBCs and hemoglobin (Supplemental Fig. 4 A). Additionally, CBC analysis following 8 weeks post transplantation of 6-month-old *Taz*^*D75H*^♂ or *wt*♂ bone marrow revealed persistent reduction of absolute lymphocyte numbers along with anemic features; including reduced RBCs, hemoglobin, and hematocrit in *Taz*^*D75H*^♂ transplants compared to unaffected *wt*♂ bone marrow transplants (Supplemental Fig. 4B). As *Taz*^*D75H*^♂ adult lymphocyte frequencies are low, there is enrichment of relative neutrophil frequency (% not absolute numbers), confirming that lymphopenia is the chief adult *Taz*^*D75H*^♂ hematopoietic defect. Together, these findings confirm that the *Taz*^*D75H*^ mutation impairs both granulopoiesis and neutrophil maturation in vivo, with early transient disruption of neutrophil morphogenesis associated with mouse age, which is reflective of the phenotype observed in some BTHS patients [2, 5, 22, 23, 38].

### Mitochondrial Dysfunction in Hematopoietic Cells from Taz^D75H^♂ Mice

**Fig. 4:**
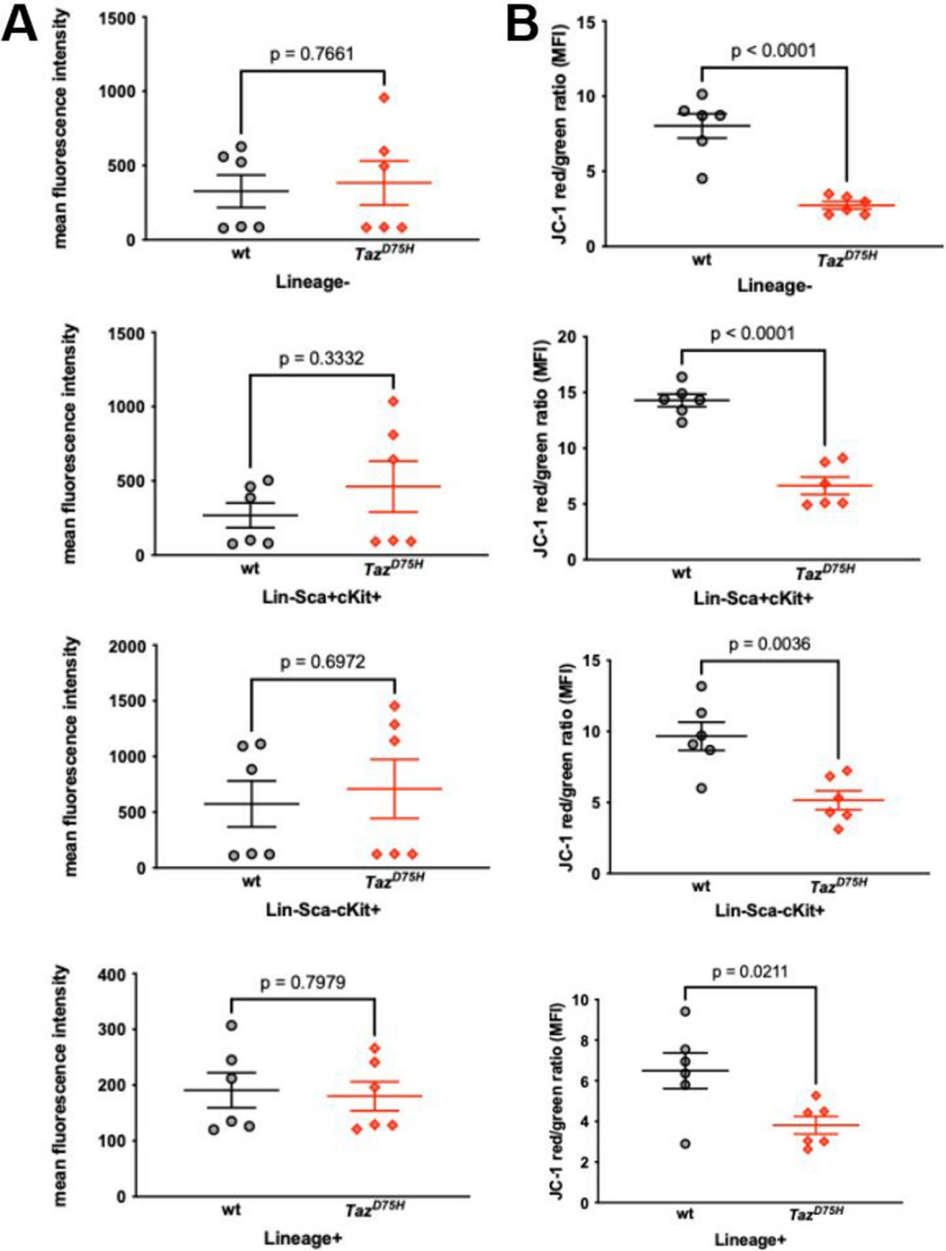
Impaired mitochondrial function in *Taz*^*D75H*^♂ bone marrow. (**A**, **B**) Flow cytometry assessment of the mitochondrial content (MitoTracker Red fluorescence intensity; A) and membrane potential (JC-1 red/green fluorescence intensity ratio; B) of hematopoietic cells derived from the bone marrow of 4-week-old *Taz*^*D75H*^♂ and *wt*♂ mice. The graphs present pooled data (means ± SEMs) from two independent experiments (*n* = 3 mice of each genotype/experiment); *p* values were calculated via *t-tests*

Next, we sought to determine the molecular phenotype underlying the impaired granulopoiesis observed in *Taz*^*D75H*^♂ mice. As mitochondrial defects are a hallmark of BTHS [3, 5] and as TAZ itself impacts many aspects of mitochondrial structure and function [1, 39, 40], we examined the mitochondrial content and membrane potential (Δψm) across HSC and HPC populations in *Taz*^*D75H*^♂ and *wt*♂ mice. Like *Taz*^*D75H*^♂ heart, skeletal muscle and livers [13], the Taz^D75H^ protein is expressed at equivalent steady-state levels to *wt*♂ in adult bone marrows (Supplemental Fig. 5 A). Flow cytometry analysis of live MitoTracker Red-stained 4-week-old ♂ bone marrow hematopoietic cells revealed statistically comparable mitochondrial content in *Taz*^*D75H*^♂ and *wt*♂ mouse lineage-negative (LSK and LK) and lineage-positive cells (Fig. 4A). However, JC-1 red/green fluorescence analysis of the mitochondrial Δψm revealed that *Taz*^*D75H*^♂ versus *wt*♂ mouse bone marrow hematopoietic cells presented a significant decrease in all the mutant cell populations examined (Fig. 4B). As the mitochondrial Δψm reflects the overall health and function of mitochondria [41], our finding that mitochondrial content is unperturbed in *Taz*^*D75H*^♂ mice but that mutant mitochondria are depolarized is indicative of impaired mutant mitochondrial function. Moreover, the finding that the mitochondrial Δψm of mutant lineage-negative cells is also reduced reinforces our hypothesis that the *Taz*^*D75H*^ defect likely occurs at the stem cell level.

**Fig. 5:**
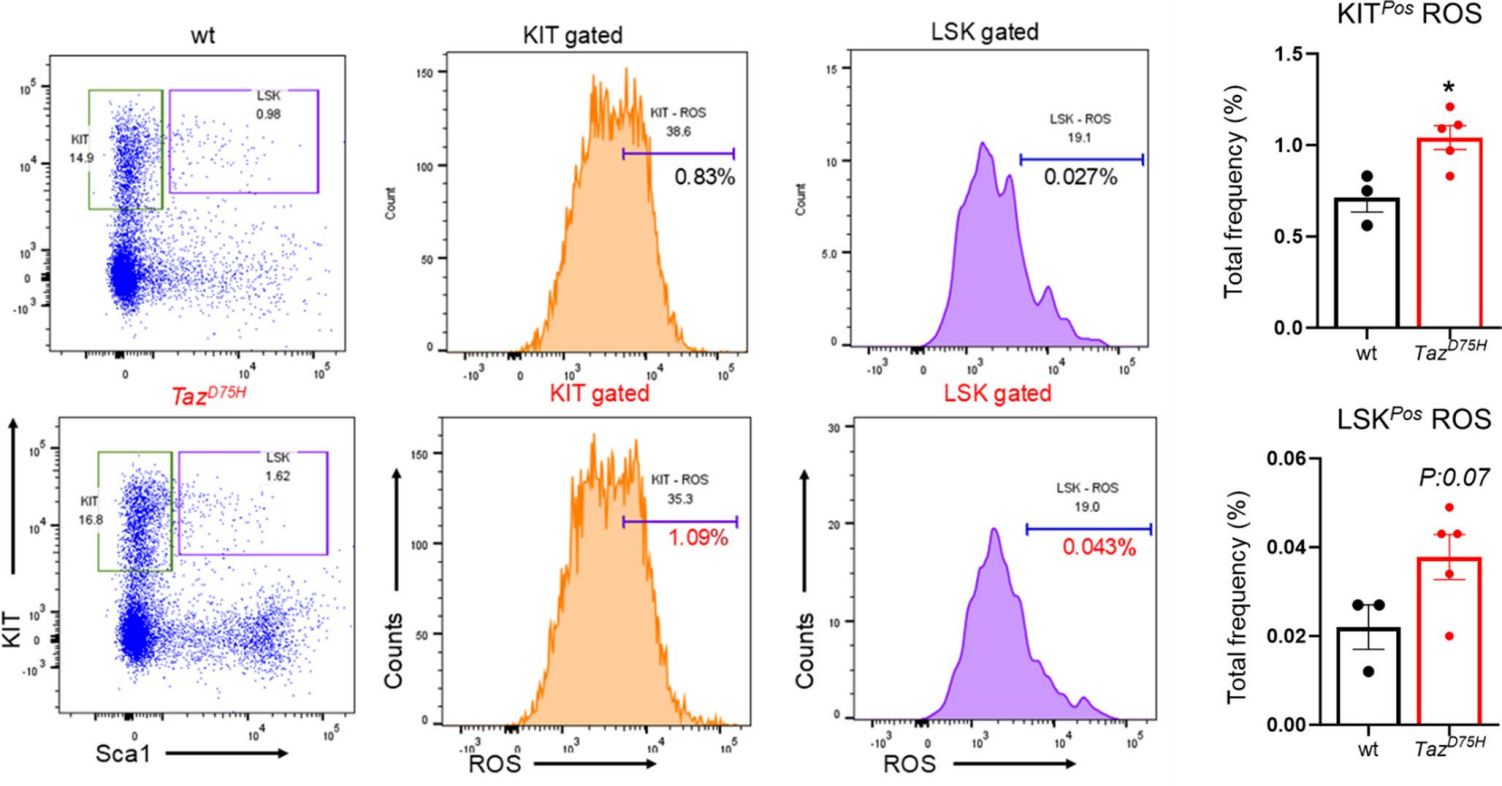
Significant accumulation of ROS in *Taz*^*D75H*^♂ myeloid progenitors. Flow cytometric analysis of ROS levels in hematopoietic cells at 8 weeks post-bone marrow transplantation of adult *wt*♂ and *Taz*^*D75H*^♂ mice. Representative flow cytometry profiles illustrate ROS levels within bone marrow myeloid progenitor (LK or KIT^Pos^) and LSK (Lin^Neg^ Sca-1^Pos^ c-Kit^Pos^) populations. Quantitative analysis is shown in the accompanying graphs, with data expressed as mean ± SEM. (*n* = 3–5 mice per group. *p ≤ 0.05)

To directly assess myeloid progenitor function, we transplanted 2 million bone marrow cells from either *Taz*^*D75H*^♂ or *wt*♂ donor mice into lethally irradiated C57BL/6 recipients and monitored the development of BTHS–associated hematologic features (Fig. 5). Peripheral blood analysis at 8 weeks post-transplant revealed an increase in circulating neutrophils and monocytes percentages along with a reduction in erythroid parameters including RBCs, hemoglobin and hematocrit in *Taz*^*D75H*^♂ marrow compared to *wt*♂ marrow (Supplemental Fig. 4). We then assessed ROS levels within bone marrow progenitor populations and found that LK (lineage negative c-Kit positive) myeloid progenitors from *Taz*^*D75H*^♂ recipients exhibited significantly elevated ROS levels (Fig. 5). Notably, *TAZ*^*D75H*^ patient CBCs confirm G-CSF-responsive neutropenia and a relative (% increase) monocytosis. Further, our *TAZ*^*D75H*^ patient has an intermittent mild microcytic anemia of unclear etiology but could be due to iron deficiency, chronic disease, or marrow dysfunction (Table 1). Combined these clinical data confirm granulopoietic dysregulation and are suggestive of anemia for those with the *TAZ*^*D75H*^ mutation.

**Table 1:** Complete blood count and differential for patients with BTHS

	Reference range + units	Patient TAZ^D75H #^	Patient A	Patient B	Patient C
Age	Years	14–16 ♂	39 ♂	42 ♂	43 ♂
G-CSF therapy		Yes	No	No	Yes
WBC	4.5–11.00 k/cumm	4.66 (3.6-6)	4.03	3.25	5.1
RBC	4.5–5.9 10^6^/cumm	5.11 (4.76–5.80)	4.45	5.77	5.75
Hb	13.9–16.3 g/dL	12.82 (11.9–14.5)	14.1	13.7	16.8
HCT	41–53%	37.98 (35.7–42.3)	43.6	45.7	51.8
Platelets	150–350 k/cumm	257.5 (246–269)	129	357	120
Neutrophil %	40–70%	30.6 (11–50)	14.6	7.8	52.6
Monocyte %	2–11%	19.5 (14–29)	42.7	23.4	8.2
Lymphocyte %	24–44%	42.87 (37–52)	35.2	53.8	30.2
Eosinophil %	1–4%	4.62 (3–8)	6	13.2	7.6
Neutrophil absolute	1.5–7.8 k/cumm	1.51 (0.4–2.7)	0.59	0.25	2.68
Lymphocyte absolute	1.1–4.80 k/cumm	1.94 (1.7–2.7)	1.42	1.75	1.54
Monocyte absolute	0.1–1.2 k/cumm	0.8 (0.6–1.1)	1.72	0.76	0.42
Red Cell Distribution Width (RDW)	11.5–14.5%	16.25 (15.4–17.2)	11.7	19.6	13.7
Mean Corpuscular Volume (MCV)	81–99 fL	74.75 (73–76)	98.0	79.2	90.1
Mean Corpuscular Hemoglobin (MCH)	27.0–34.0 pg	25.25 (24.9–25.4)	31.7	23.7	29.2

Each BTHS patient has been genetically confirmed to have pathogenic variants in *TAFAZZIN* and elevated plasma MLCL/CL ratios. Our *TAZ*^*D75H*^ patient (^#^) exhibits G-CSF-responsive neutropenia, a relative monocytosis and a mild intermittent microcytic anemia. Data represent the average (with *TAZ*^*D75H*^ ranges included) of eight CBC results over two years. Thrombocytopenia is present in patients A and C, and low-normal lymphocyte counts are present in patients A-C

### Cyclosporine Rescues Mitochondrial Damage in Patient TAZ^D75H^ Lymphoblasts

**Fig. 6:**
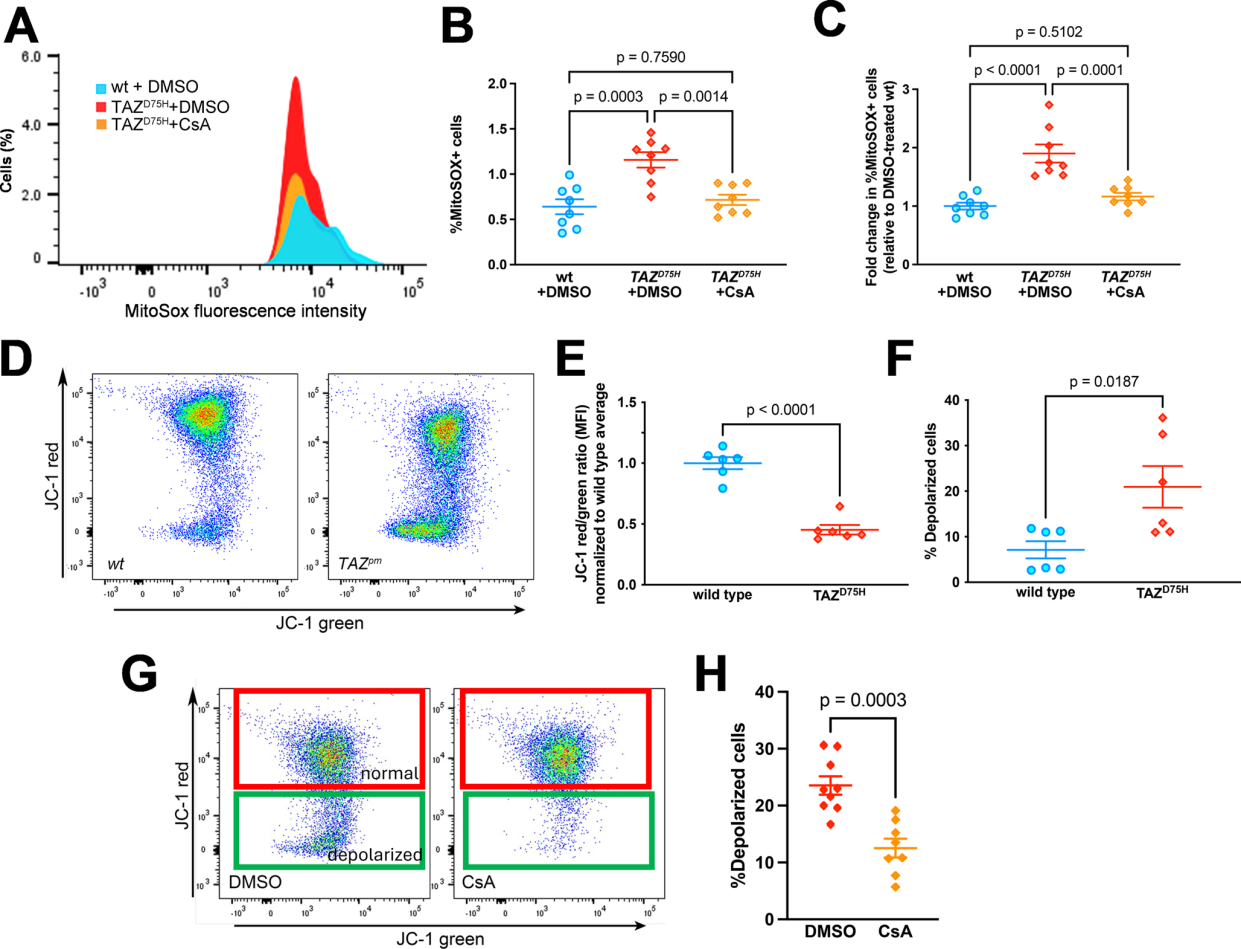
Cyclosporine suppresses superoxide production and restores mitochondrial membrane potential in BTHS patient lymphoblasts. (**A**) Representative flow cytometry histograms and quantification (**B**) of MitoSOX-positive populations in patient *TAZ*^*D75H*^ and control lymphoblasts treated with DMSO versus *TAZ*^*D75H*^ lymphoblasts treated with CsA (10µM). (**C**) Quantification of fold-change in the percentage of MitoSOX-positive cells detected by flow cytometry. Data is from two independent experiments, where *n* = 8 per condition. P values were calculated by one-way ANOVA. (**D**) Representative flow cytometry dot plots and quantification (**E**) of red versus green emission in JC-1-stained control and *TAZ*^*D75H*^ lymphoblasts. (**F**) Quantification of percent depolarized (i.e. percentage of cells positive for green JC-1 fluorescence) lymphoblasts. (**G**) Representative flow cytometry dot plots of red versus green emission in JC-1-stained *TAZ*^*D75H*^ lymphoblasts treated with DMSO or CsA. (H) Quantification of percent depolarized (i.e. percentage of cells positive for green JC-1 fluorescence) *TAZ*^*D75H*^ lymphoblasts after treatment with DMSO or CsA (10µM). Data is from two independent experiments (*n* ≥ 8 technical replicates/condition). *P* values were calculated by t-test

To confirm that mitochondrial dysfunction is secondary to the *TAZ*^*D75H*^ mutation in BTHS neutrophils and their progenitors, we utilized immortalized lymphoblasts derived from our BTHS patient (*TAZ*^*D75H*^♂ cells). Significantly, the TAZ^D75H^ protein is expressed in *TAZ*^*D75H*^♂ cells at comparable levels to wildtype TAFAZZIN in control EBV-immortalized human male lymphoblasts (Supplemental Fig. 5B). Importantly, TEM analysis of these cells revealed abnormally disorganized swollen and empty mitochondria (Supplemental Fig. 6), irregular perinuclear clustering, and mitochondrial clumping, indicating a stress response and/or mitochondrial dysfunction [42]. Consistent with these findings, *TAZ*^*D75H*^♂ lymphoblasts exhibited a trend of reduced expression of select electron chain transport complexes (Supplemental Fig. 7). Furthermore, flow cytometry analysis revealed elevated mitochondrial superoxide and loss of mitochondrial Δψm in *TAZ*^*D75H*^♂ versus control lymphoblasts (Fig. 6). Indeed, extensive studies across various murine models have demonstrated that even modest increases in mitochondrial ROS, often in the range of 1.2–1.5-fold above baseline, are sufficient to induce measurable mitochondrial dysfunction. This includes loss of Δψm, mtDNA damage, altered mitochondrial dynamics, and skewing of stem or progenitor cell fate [43, 44]. Thus, even small subsets of MitoSOX⁺ cells or subtle ROS elevations in our model are biologically meaningful, especially when corroborated by structural mitochondrial damage and molecular anomalies as observed in our TEM and Western analysis (see Supplementary Figs. 6 and 7).

Under oxidative stress, the mitochondrial matrix-resident cyclophilin D (CypD) translocates to the IMM, opening the mitochondrial permeability transition pore (mPTP). This phenomenon rapidly increases the permeability of the IMM, resulting in mitochondrial swelling and membrane depolarization, potentiating cell death [45–49]. The mPTP‒CypD signaling axis is activated by ROS in HSCs, and this pathway has been successfully targeted to increase life span in a mouse model of lethal mitochondrial myopathy via the clinically approved small molecule inhibitor Cyclosporine A (CsA) [50]. CsA binds and inhibits CypD, prohibiting mPTP induction [51, 52]. To evaluate whether CsA could normalize superoxide levels and the mitochondrial Δψm in BTHS, we treated *TAZ*^*D75H*^♂ lymphoblasts with CsA (or DMSO as a control) and assayed the mitochondrial superoxide levels and Δψm through MitoSOX and JC-1 staining, respectively. Flow cytometry analysis revealed that exposure to CsA reduced TAZ^D75H^♂ cell mitochondrial superoxide to control levels (Fig. 6A–C; Supplemental Fig. 8) and reduced the percentage of *TAZ*^*D75H*^♂ cells with mitochondrial membrane depolarization (Fig. 6G, H). These results suggest that CsA, and potentially other pharmacological agents that target the mPTP-CypD axis, may be therapeutically beneficial for treating BTHS hematopoietic defects.

## Discussion

In this study, we demonstrated that surviving patient-tailored knock-in mutant *Taz*^*D75H*^♂ mice, which recapitulate the major BTHS-associated phenotypes [13, 53], exhibit impaired granulopoiesis and early-onset neutropenia. Using CBC analysis and ex vivo/in vivo HPC and hematopoietic cell analyses, we found that *Taz*^*D75H*^♂ mice exhibit an age-dependent transient impairment of juvenile neutrophil maturation and persistent lymphopenia. Notably, we also demonstrated that myeloid progenitor cells from juvenile *Taz*^*D75H*^♂ mice are functionally abnormal. Specifically, both mutant lineage-negative (LSK and LK myeloid progenitors) and lineage-positive bone marrow cells exhibit decreased mitochondrial membrane potentials, which likely impairs energy production and signaling pathways. Importantly, this confinement to the neonatal and juvenile stages is consistent with reports that BTHS neutropenia can be observed before birth [22]. Furthermore, the lack of neutropenia and absence of changes in *Taz*^*D75H*^♂ neutrophil CD45.2^+^ percentages in 10-month-old *wt*♂ transplanted mice confirmed a juvenile-restricted effect and a similar adult mutant reconstitution potential. As neutrophils have a brief lifespan [25], our observations in the *Taz*^*D75H*^♂-cell transplanted *wt*♂ model suggest that cell-autonomous changes within the *Taz* gene underlie the observed *Taz*^*D75H*^♂ granulopoiesis defects.

The *Taz*^*D75H*^ model results indicate a unique granulopoiesis phenotype distinct from that associated with existing *Tafazzin* alleles. Notably, in contrast to all the other *Tafazzin* alleles, the *Taz*^*D75H*^ allele lacks acyltransferase activity but expresses a stable mutant protein, resulting in a buildup of immature MLCL relative to CL in the *Taz*^*D75H*^♂ mouse circulation [13]. In previous studies using the doxycycline-inducible transgenic *Tafazzin shRNA*-mediated model [54, 55], in which wildtype Tafazzin levels are reduced via doxycycline feeding in the *in utero* and postnatal stages, 50% of the 2-month-old *Taz*^*KD*^♂ mice demonstrated neutropenia, as did 20% of the *wt* controls (with doxycycline administered to both). Similarly, an in vitro analysis of bone marrow mesenchymal stem cells from 3–5-month-old *Taz*^*KD*^♂ mice revealed that increased ROS generation and glycolysis can promote activated B lymphocyte reprogramming [56]. However, when *Tafazzin shRNA*-knockdown mice were fed doxycycline at 7.6–14.3 weeks of age, neutrophil numbers were unaffected in adult *Taz*^*KD*^♂ mice despite an abnormal increase in the MLCL/CL ratio [57]. Given that doxycycline itself can impair mitochondrial function [55] and decrease the number of circulating neutrophils [58], the distinct *Taz*^*KD*^♂ phenotype likely confirms that Tafazzin knockdown affects neutrophils only when administered *in utero*/neonatally [56]. These data support a Taz-specific requirement for juvenile neutrophil morphogenesis.

Conversely, null *Taz*^*KO*^♂ mice that systemically lack Tafazzin [36] demonstrate reduced circulating neutrophil numbers at 6 months, whereas the neonatal/juvenile hematopoietic status remains unknown. Moreover, the use of the estrogen receptor-Hoxb8 fusion system to conditionally immortalize GMPs from *Taz*^*KO*^♂ and *wt* mouse E14-E16 fetal livers [21] revealed that null myeloid progenitor development was largely unaffected; however, the transplantation of null fetal liver cells caused mild and consistent leukopenia. Likewise, the absence of Tafazzin in *Taz*^*KO*^♂ fetal livers did not cause any significant differences in neutrophil development or function, including phagocytosis, cytokine production, or ROS [21]. Whether the various *Tafazzin* allele differences in neutrophil phenotypes are reflective of inherent BTHS variability, a subset of BTHS patients, or are due to lower wildtype Tafazzin levels, a complete absence of Tafazzin and/or the presence of a stable Taz^D75H^ mutant protein remains to be determined. Significantly, characterization of *TAFAZZIN* variants demonstrated BTHS-causing mutations frequently result in expression of stable mutant proteins with variable phenotypes [11, 12, 18]. Thus, BTHS is not solely driven by loss of TAFAZZIN protein. Additionally, TAFAZZIN can sense IMM curvature and its hydrophilic domain (which is intact in the Taz^D75H^ protein) is known to bind complexes containing ADP/ATP-carrier and -synthase, the Prohibitin/Dnajb6 complex, and drives glycerolipid catalysis [4, 14–17]. Suggestively, while *Taz*^*D75H*^♂ hearts exhibit dysregulated p53/p53-target, autophagic AMPK/LC3B and senescent p16 signaling variations, none of these effectors are mis-expressed in *Taz*^*KO*^♂ hearts [13]. Given TAFAZZIN has additional non-enzymatic functions and there are known signaling disparities between the alleles, these findings suggest the TAZ^D75H^ mutant protein retains partial function, conferring pathogenicity through a distinct mechanism-of-action.

Although the mechanism underlying the age-specific presentation of neutropenia remains uncertain, declining CL content in aged versus young HSCs [59] could indicate that CL is less essential during adult neutrophil replenishment. Aging itself has also been shown to alter neutrophil production and the ability of neutrophils to respond to infections [60]. Additionally, the spatiotemporal-restricted requirement of *Tafazzin* in HSCs, HPCs, lymphoblasts, and neutrophils remains unknown, allied to the finding that cardiomyocyte-restricted conditional knockouts of *Tafazzin* are surprisingly viable [36]. Further examination of the role of impaired granulopoiesis in *Tafazzin* allele-associated partial *in utero*/newborn mutant lethality [13, 36, 37] and in prenatal BTHS is warranted [5]. Likewise, as fetal HPCs can respond to prenatal inflammation *in utero*, and the fetal response can shape postnatal hematopoiesis and immune cell function [61, 62], it remains to be determined whether neonatal/juvenile impairment of mutant neutrophil maturation may drive subsequent WBC/lymphogenic alterations in *Taz*^*D75H*^♂ adults. Although neutropenia is the primary BTHS hematologic disorder, both monocytosis and dysregulated bone marrow promyelocyte-myelocyte maturation are also observed in BTHS [20, 23, 24], and there is a report of BTHS-associated B cell lymphopenia and hypogammaglobulinemia [38]. Moreover, anemia has also been reported [63, 64] even though the number of published cases in BTHS are currently limited. Significantly, bone marrow transplantation data confirmed aberrant *Taz*^*D75H*^♂ myeloid progenitor expansion leading to increased circulating neutrophils and monocytes percentages along with a reduction of RBCs, hemoglobin and hematocrit (*see* Fig. 5). Notably, the *TAZ*^*D75H*^ patient exhibits neutropenia, monocytosis and iron deficiency anemia (Table 1). Additionally, we identified three adult BTHS patients, two who were neutropenic and one receiving G-CSF treatment, whose lymphocyte counts are at the “low end of normal” and two were thrombocytopenic (Table 1). Therefore, we suggest that comprehensive immunology evaluation of BTHS patients is likely to reveal a constellation of hematological findings and may offer valuable phenotype-genotype information and insights.

To investigate the role of TAFAZZIN enzymatic activity in BTHS hematopoietic cells, we examined the mitochondrial structure and function of immortalized TAZ^D75H^♂ lymphoblasts. Like the *Taz*^*KO*^♂ mouse [13] and BTHS patient cells [65], *TAZ*^*D75H*^♂ lymphoblasts exhibited ultrastructural cristae defects and abnormally swollen mitochondria. Furthermore, *TAZ*^*D75H*^♂ lymphoblast mitochondria were functionally affected and displayed increased superoxide levels and reduced mitochondrial membrane potential, both indicators of mitochondrial stress. Indeed, an absence of CL from the IMM can drive electron leakage from the electron transfer system, defective NADPH oxidative stress signaling, and reduced mitochondrial Δψm in BTHS [65–67]. Importantly, BTHS bone marrow neutrophils demonstrate impaired differentiation and maturation, whereas circulating BTHS neutrophils demonstrate an immature phenotype and a dysregulated neutrophil inflammatory response [24]. Similarly, *shRNA*-mediated TAFAZZIN knockdown in several leukemic cell lines is sufficient to alter stemness and differentiation and induce cell cycle arrest but not apoptosis [57]. Further, BTHS lymphoblasts lack a mature CL and present increased superoxide anion levels and loss of mitochondrial Δψm [28, 65]. As we found that CsA (an immunosuppressant) can restore *TAZ*^*D75H*^♂ lymphoblast mitochondrial potential and reduce superoxide levels, this suggests beneficial antioxidant and functional mitochondrial effects. Furthermore, CsA can prevent mitochondrial swelling and membrane depolarization by targeting mitochondrial CypD, a key component in mPTP opening, leading to mitochondrial dysfunction [45–49]. Although the hematopoietic status of CypD expression is uncertain, CypD can contribute to normal neutrophil activation [68] and is upregulated in *Taz*^*KD*^♂ mice [69]; thus, these data suggest that therapeutic targeting of the mPTP‒CypD axis may be beneficial. Indeed, elamipretide (a mitochondrial-targeting peptide) is currently used to treat BTHS [6, 70], as it inhibits mPTP opening, prevents oxidative damage, and helps maintain cristae integrity. Future studies will be needed to directly test whether CsA reverses TAZ^D75H^-mediated neutropenia in vivo or in an ex vivo neutrophil culture system. Taken together, these data support our hypothesis that a lack of TAFAZZIN acyltransferase activity is sufficient to phenocopy BTHS neutropenia and drive mitochondrial dysfunction, demonstrating that normal granulopoiesis requires CL phospholipids.

Interestingly, the apparent reduction in myeloid progenitor colony formation *in vitro (see* Fig. 2*)*, despite preserved in vivo output (*see* Fig. 1), points to a context-dependent functional defect that is likely influenced by cytokine signaling or the microenvironment, rather than intrinsic depletion or only dysfunction of progenitor populations. This suggests that the myeloid progenitors in *Taz*^*D75H*^♂ marrow remain functionally competent in vivo due to the presence of essential regulatory cues derived from stromal signals, cell-cell interactions, and finely tuned cytokine gradients, which may not adequately replicate in vitro colony assays. Alternatively, the in vitro myeloid progenitor assay may recapitulate aspects of stress hematopoiesis which are required for the deficiency in progenitors and not present in the transplant model. A particularly relevant factor in this context is IL-6, a pleiotropic cytokine, which is shown to be consistently elevated in the serum of BTHS patients [71]. IL-6 primarily induces differentiation and suppresses proliferation. Sustained IL-6 exposure can induce promote ROS generation, mitochondrial dysfunction, HSPC exhaustion, and drive pathological remodeling of the niche [72, 73]. In vivo, the hematopoietic niche may regulate the effects of elevated IL-6 through compensatory anti-inflammatory pathways or niche-protective mechanisms, thereby preserving baseline myelopoiesis [74–76]. However, under in vitro conditions, where cells are removed from their native regulatory microenvironment and exposed to cytokine cocktails this buffering is absent, reflecting a more stressed scenario and indicate that the defect in GMPs may require specific stressors to elicit the phenotype. As a result, the suppressive influence of IL-6 may become unmasked, leading to impaired colony-forming capacity, as higher IL-6 in vivo could also help the mice overcome the myeloid progenitor deficiency since it drives myeloid differentiation. We speculate that excessive IL-6 signaling might be a key contributor to the observed discrepancies between in vitro functional effect on CFU and in vivo phenotypic expression of progenitors seen *Taz*^*D75H*^♂ bone marrow cells. Conversely, the high levels of IL-6 in vivo may promote myelopoiesis and increase progenitors thereby circumventing the deficiency in myeloid progenitors observed in vitro.

Although several groups have investigated the mechanisms by which *TAFAZZIN* mutations result in BTHS-related cardiac and skeletal muscle defects [36, 37, 55, 77], the reason BTHS patients have fewer neutrophils remains unclear. Our data demonstrate a novel stage-restricted defect within mouse and patient *D75H-*mutant neutrophil maturation associated with deficient hematopoietic progenitor differentiation, abnormal depolarization of mutant hematopoietic cell mitochondrial membranes, and ROS buildup. One limitation of our study is the lack of serial CBC measurements from individual mice over time. A longitudinal study of absolute cell counts from individual mice will be necessary to detect periodic neutropenia. Future studies are also needed to assess whether *TAZ*^*D75H*^ HSCs can function appropriately via in vivo competition assays and transplantation into secondary hosts to confirm whether the initial defect is at the level of stem cells. Additionally, the functions of both reduced neonatal/juvenile and unchanged adult *TAZ*^*D75H*^ neutrophils need to be examined for their bacterial killing capacity and NET formation ability [78]. Also, as some BTHS patients and *Taz*^*D75H*^♂ exhibit 3-methylglutaconic aciduria [5, 7, 13], the effect of elevated 3-methylglutaconic upon granulopoiesis warrants further investigation. In summary, our work demonstrated that impaired hematopoiesis in BTHS patients can be recapitulated in vivo in mice by editing a patient-specific *TAFAZZIN* point mutation into the murine genome. This patient-tailored *Taz*^*D75H*^♂ mouse model recapitulates the clinical hallmarks of BTHS neutropenia and demonstrates other hallmark BTHS phenotypes in nonhematopoietic tissues [13, 53], accompanied by structural and functional mitochondrial defects. This work enables future in vivo studies to enhance our mechanistic understanding of hematopoietic defects in BTHS and develop treatments.

## Supplementary Information

Below is the link to the electronic supplementary material.

Supplementary Material 1

## Data Availability

No datasets were generated or analysed during the current study.
