# Correction to: Granulopoietic Dysregulation in a Patient-Tailored Mouse Model of Barth Syndrome

**DOI:** 10.1007/s12015-025-10983-9

**Published:** 2025-09-30

**Authors:** Elizabeth A. Sierra Potchanant, Maegan L. Capitano, Donna M. Edwards, Baskar Ramdas, Scott Cooper, James Ropa, S. Louise Pay, Aditya Sheth, Paige L. Snider, Hilary J. Vernon, Ngoc-Tung Tran, Reuben Kapur, Simon J. Conway

**Affiliations:** 1 Department of Pediatrics, Herman B. Wells Center for Pediatric Research, Indiana University School of Medicine, 1044 West Walnut Street, Indianapolis, IN 46202 USA; 2 Department of Microbiology & Immunology, Indiana University School of Medicine, 635 Barnhill Drive, Indianapolis, IN 46202 USA; 3 Department of Medical & Molecular Genetics, Indiana University School of Medicine, 410 West 10th Street, Indianapolis, IN 46202 USA; 4 Stark Neurosciences Research Institute, Indiana University School of Medicine, 320 West 15th Street, Indianapolis, IN 46202 USA; 5 Department of Genetic Medicine, Johns Hopkins University School of Medicine, 733 North Broadway, Baltimore, MD 21205 USA

**Correction to: Stem Cell Reviews and Reports (2025) 21:2170–2187**

10.1007/s12015-025-10945-1

The original published version of this article unfortunately contained a mistake.

 In this article, Simon J. Conway should also be denoted as a corresponding author. The error occurred during the typesetting and proofing process.

The original article has been corrected.

