# Supplementary material for: Granulopoietic Dysregulation in a Patient-Tailored Mouse Model of Barth Syndrome
Source: Stem Cell Rev Rep. 2025 Aug 5;21(7):2170–87. doi: 10.1007/s12015-025-10945-1 (PMC12408712; doi:10.1007/s12015-025-10945-1)
Supplement: Supplementary file 1 — Supplementary Material 1 [file 12015_2025_10945_MOESM1_ESM.pdf]

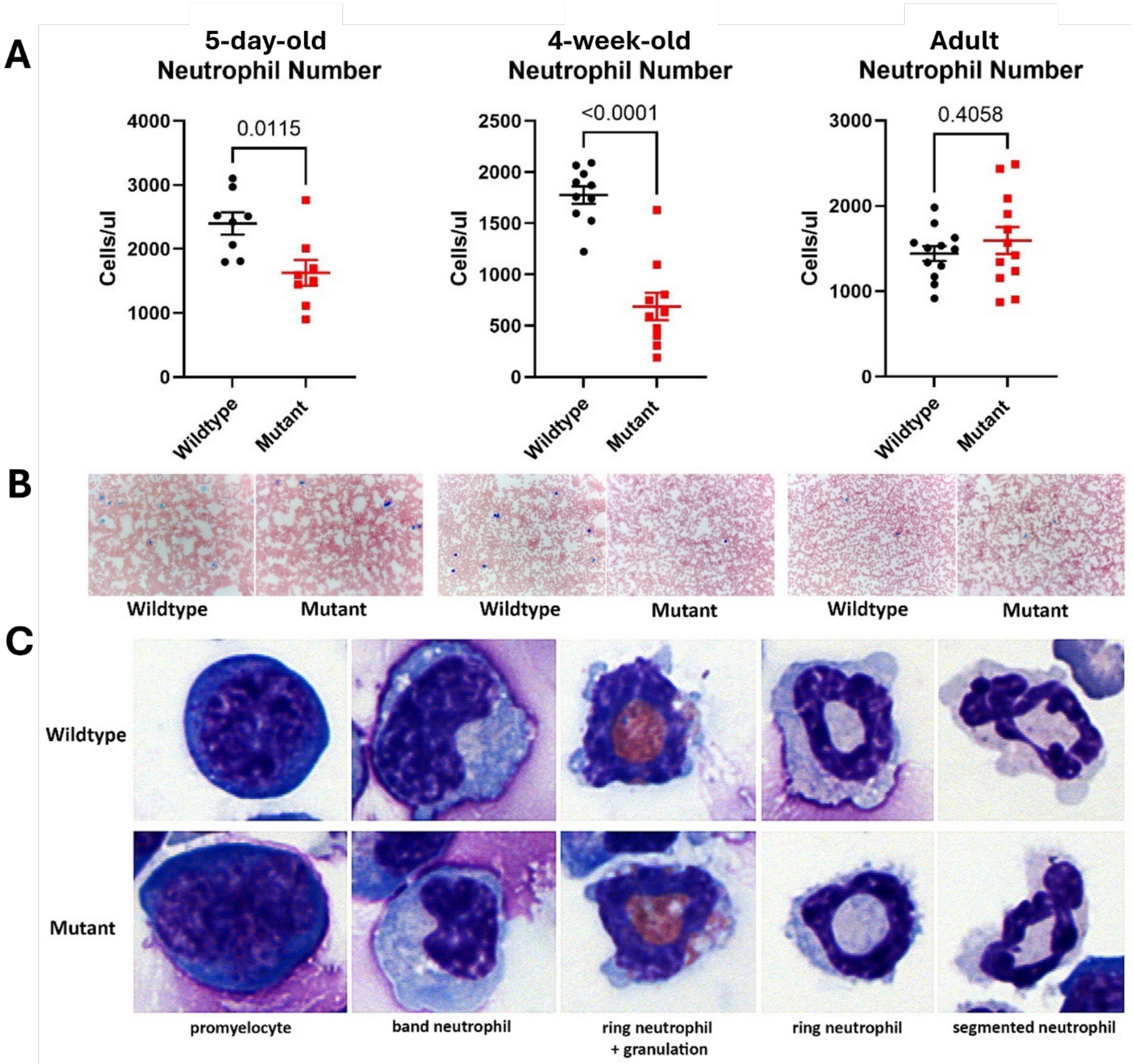

Supplemental Figure 1. Neutropenia is present only in neonatal and juvenile *Taz*^{D75H}♂ mice. (A, B) Statistical analysis (A) and representative Giemsa-stained images (B) of absolute neutrophil counts per microliter (cells/μL) in neonatal (n = 8/genotype), juvenile (n = 10/genotype) and mature adult (n = 12/genotype) *Taz*^{D75H}♂ peripheral blood samples revealing a <31% (p = 0.0115) and <59% reduction (p = 0.0001) in neonatal and juvenile *Taz*^{D75H}♂ versus *wt*♂ mouse neutrophils but no differences in those from mature adults (p = 0.4058). (C) Representative Wright–Giemsa-stained images of juvenile *Taz*^{D75H}♂ and *wt*♂ littermate bone marrow cytospin preparations at different stages of maturation (n = 4/genotype). B = x200 magnification; C = x630 magnification.

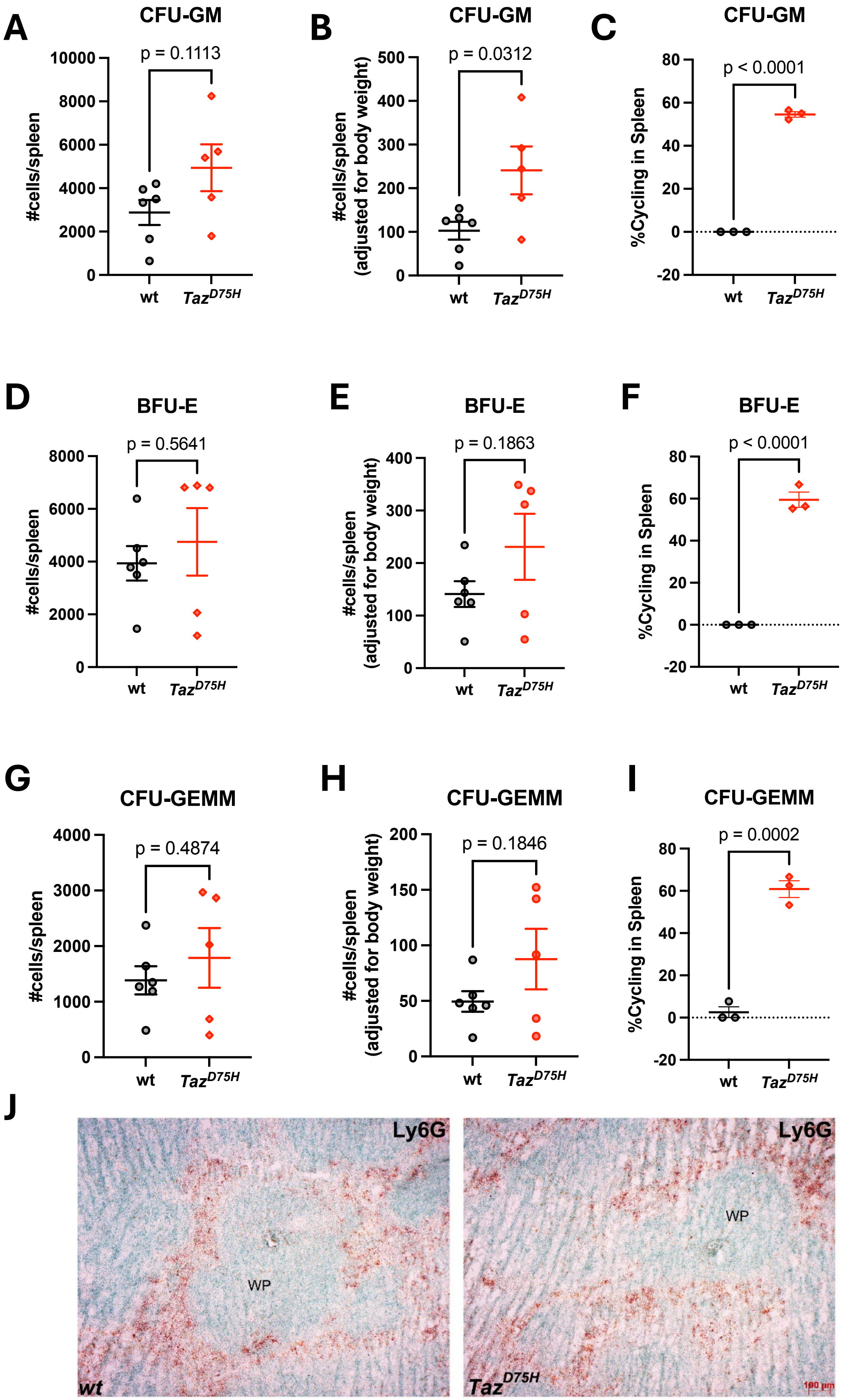

A
CFU-GM
p = 0.1113
#cells/spleen
wt
Taz D75H
B
CFU-GM
p = 0.0312
#cells/spleen (adjusted for body weight)
C
CFU-GM
p < 0.0001
%Cycling in Spleen
D
BFU-E
p = 0.5641
E
BFU-E
p = 0.1863
F
BFU-E
p < 0.0001
G
CFU-GEMM
p = 0.4874
H
CFU-GEMM
p = 0.1846
I
CFU-GEMM
p = 0.0002
J
Ly6G
WP
wt
Ly6G
WP
Taz D75H
100 µm

Supplemental Figure 2. Increased function of hematopoietic myeloid progenitors in the *Taz*^{D75H}♂ spleen. Colony-forming units (CFUs) for granulocyte-macrophage (A), burst-forming unit-erythrocyte (D), and granulocyte-erythrocyte-macrophage-megakaryocyte (G) numbers per spleen of *Taz*^{D75H}♂ and *wt*♂ mice as determined by colony assay. (B, E, H) Body weight-adjusted numbers of spleen CFUs. (C, F, I) The percentage of HPCs cycling per spleen was determined via a thymidine kill assay. For the CFU data, the graphs represent pooled data (means ± SEMs) from two independent experiments (n = 3/genotype/experiment). For the thymidine kill data, the graphs represent data from a single experiment (n = 3/genotype). (J) Lymphocyte antigen 6 (Ly6G) complex locus protein immunohistochemistry (brown immunosignal) in *wt*♂ and *Taz*^{D75H}♂ adult spleen cryostat sections counterstained with methyl green (n = 3 spleens/genotype). Abbreviation: WP, lymphoid white pulp. Scale bar = 100μm.

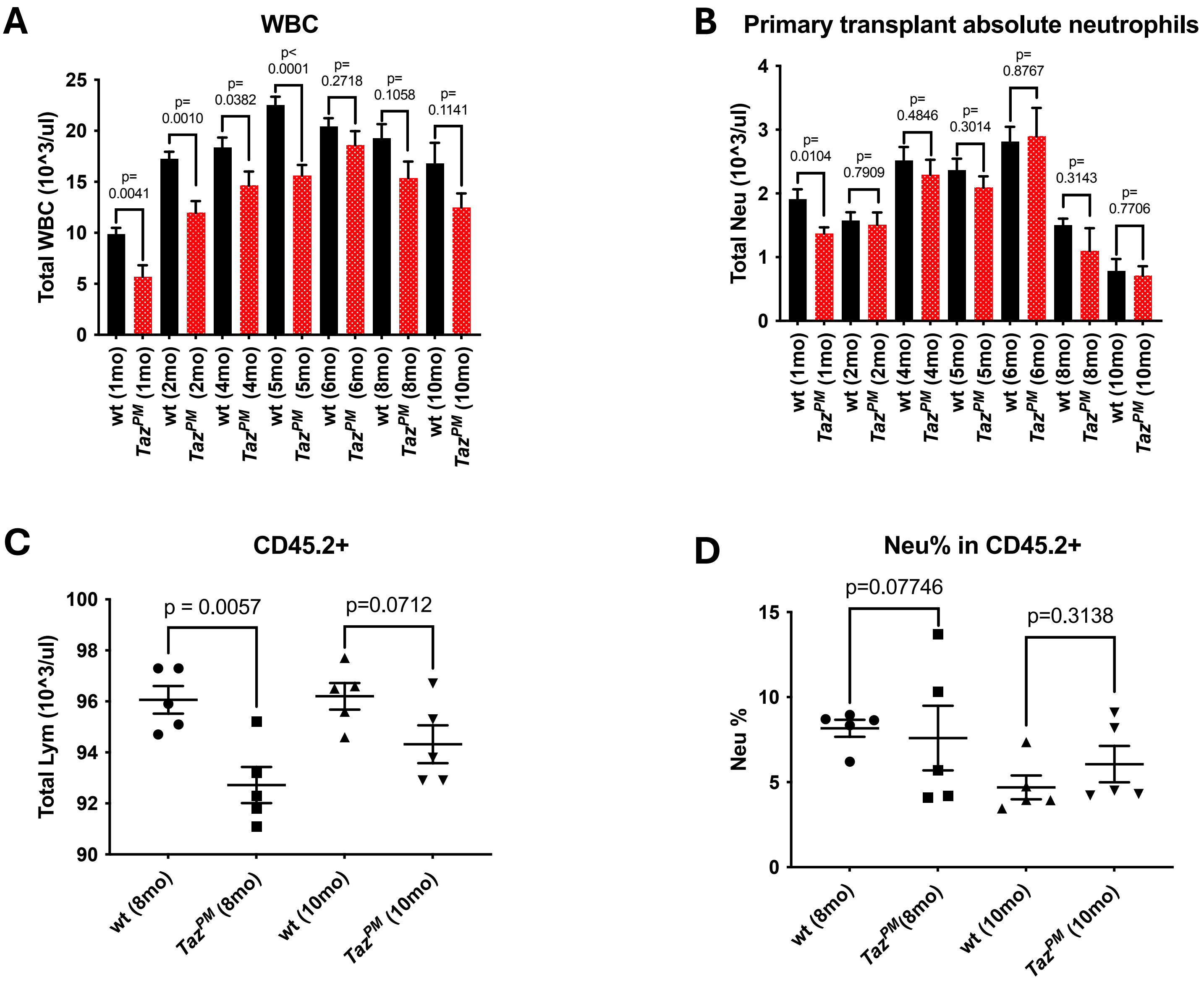

Supplemental Figure 3. The neutropenia *Taz*^{D75H} mutation induces persistent lymphopenia *in vivo*. Absolute white blood cell (A) and neutrophil (B) counts from the peripheral blood of transplanted mice at the indicated times. Quantification of lymphocyte (C) and neutrophil percentages in CD45.2+ cells from transplanted mice at the indicated times (n = 10 recipient mice/genotype).

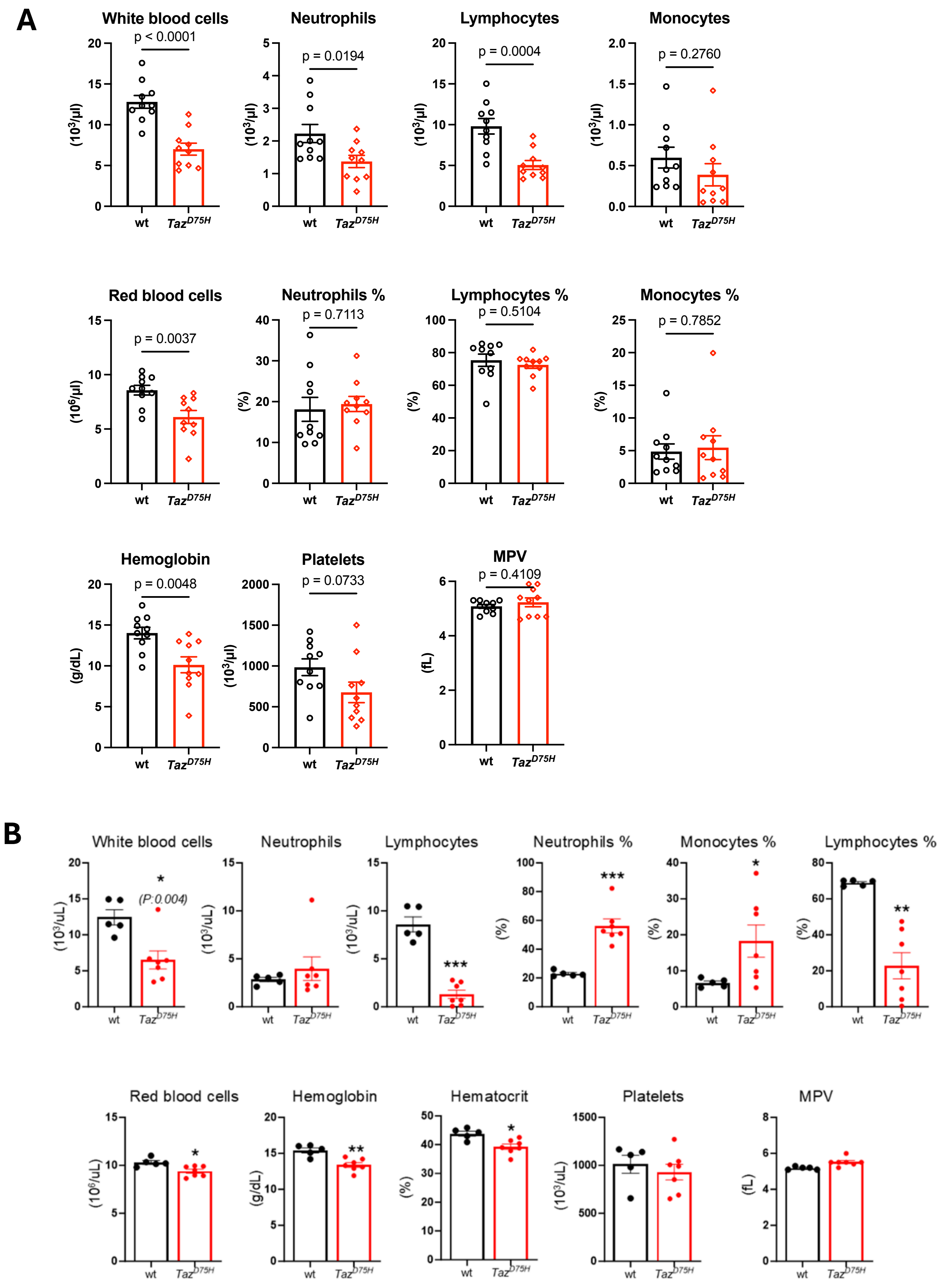

A
White blood cells
p < 0.0001
Neutrophils
p = 0.0194
Lymphocytes
p = 0.0004
Monocytes
p = 0.2760
Red blood cells
p = 0.0037
Neutrophils %
p = 0.7113
Lymphocytes %
p = 0.5104
Monocytes %
p = 0.7852
Hemoglobin
p = 0.0048
Platelets
p = 0.0733
MPV
p = 0.4109
wt
TazD75H
B
White blood cells
(P:0.004)
Neutrophils
Lymphocytes
Neutrophils %
Monocytes %
Lymphocytes %
Red blood cells
Hemoglobin
Hematocrit
Platelets
MPV

Supplemental Figure 4: *Taz*^{D75H}♂ bone marrow exhibits anemic features. (A) CBC analysis of peripheral blood from 3-5 week-old male mice of the indicated genotypes. Graphs represent means ± SEM (n=10 mice/genotype), and *p* values were calculated by t-test. (B) CBC analysis of ***wt***♂ mice transplanted with bone marrow of the indicated genotypes. CBC of peripheral blood at 8 weeks post transplantation revealed a myeloid-skewed hematopoietic profile in *Taz*^{D75H}♂ bone marrow recipients compared to *wt*♂ bone marrow recipients. Notably, *Taz*^{D75H}♂ recipients exbibit reduced WBCs and lymphocytes, in addition to elevated neutrophils compared to *wt*♂ recipients receiving *wt*♂ bone marrow. Further, anemic features of reduced RBCs, Hb and HCT was observed in *Taz*^{D75H}♂ compared to *wt*♂ bone marrow transplants. Quantitative data are presented as mean ± SEM, with *wt*♂ recipients (n=3) and *Taz*^{D75H}♂ recipients (n=5). *p≤0.05, by student`s t-test.

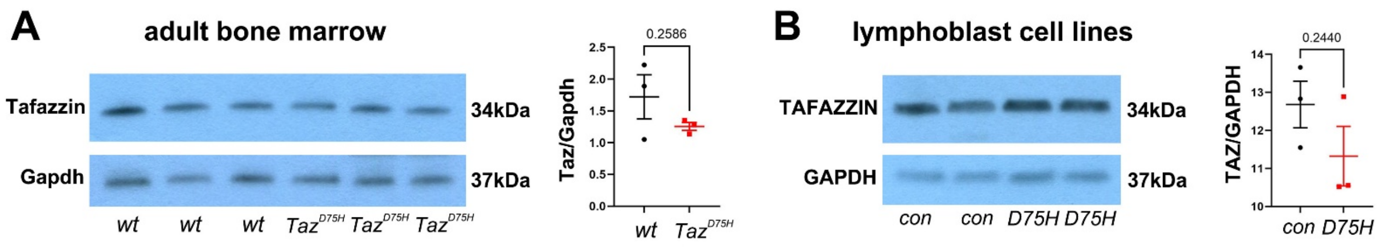

Supplemental Figure 5: Mutant Taz protein levels are not perturbed by *Taz*^{D75H} knock-in allele nor via the patient *TAZ*^{D75H} mutation. (A) Representative Western blot images of triplicate *wt*♂ and *Taz*^{D75H}♂ adult bone marrow probed for Tafazzin and Gapdh, with quantification of Tafazzin protein levels normalized to Gapdh control (n=3 samples/genotype). (B) Representative Western blot images of duplicate control♂ and *TAZ*^{D75H} lymphoblast cell lysates probed for TAFAZZIN and GAPDH, with quantification of TAFAZZIN protein levels normalized to GAPDH (n=3 samples/genotype). Data represents three technical replicates.

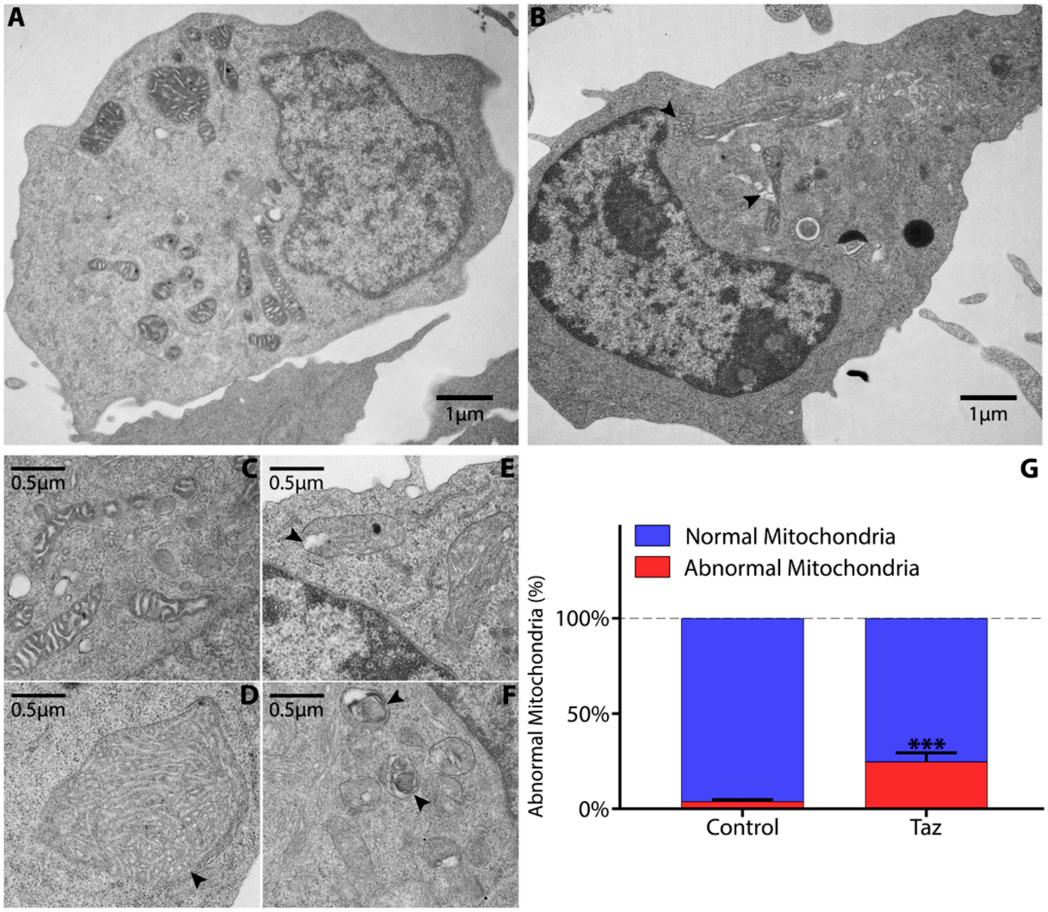

Supplemental Figure 6: *TAZ*^{D75H}♂ lymphoblasts exhibit abnormal mitochondrial ultrastructure. Representative TEM electron micrograph images of healthy control (A) and *TAZ*^{D75H} patient lymphoblasts (B) are shown. Honeycomb-like formations and electron-translucent mitochondrial vacuoles are denoted with black arrowheads (A). Representative micrographs of control mitochondria (C) along with deteriorating *TAZ*^{D75H} mitochondria with various mitochondrial defects (honeycomb-like formations, electron-translucent mitochondrial vacuoles, giant clusters and onion ring-like autophagic vacuoles) are denoted with black arrowheads (D-F). Quantitative comparisons of abnormal mitochondrial percentage is shown (G). Statistical analysis was done using student's t-test. For control cells, n=14 cells analyzed. For *TAZ*^{D75H}, n=16 cells analyzed.

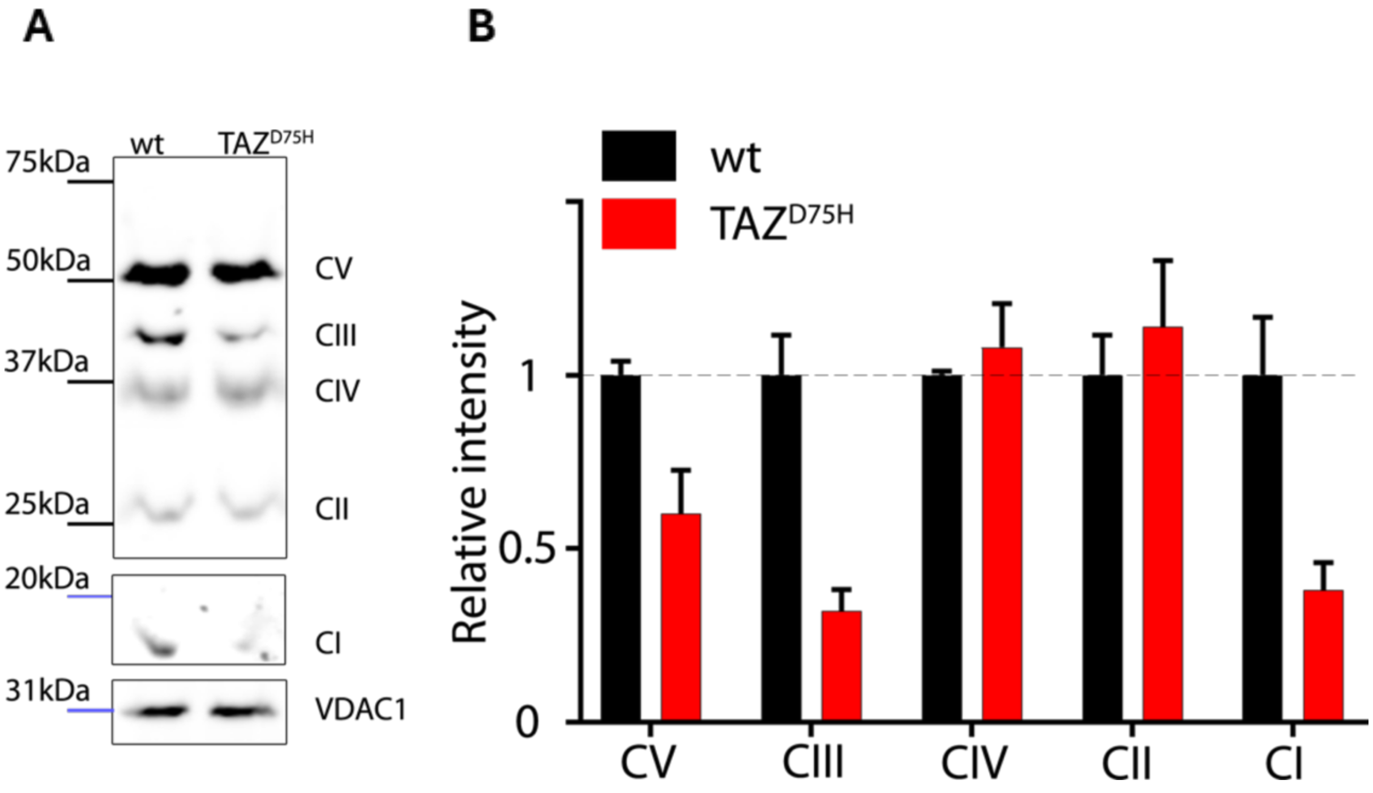

Supplemental Figure 7. ***TAZ^{D75H}*♂ lymphoblasts exhibit reduced expression of select electron transport chain complexes (ETC).** (A) Representative Western blot of purified mitochondrial lysates from human lymphoblasts. (B) Quantitative comparison of *TAZ^{D75H}* ETC complex expression, relative to control♂ lymphoblast ETC complex expression. Data is representative of two independent experiments (n= 3 technical replicates/experiment). Error bars represent standard error of the mean.

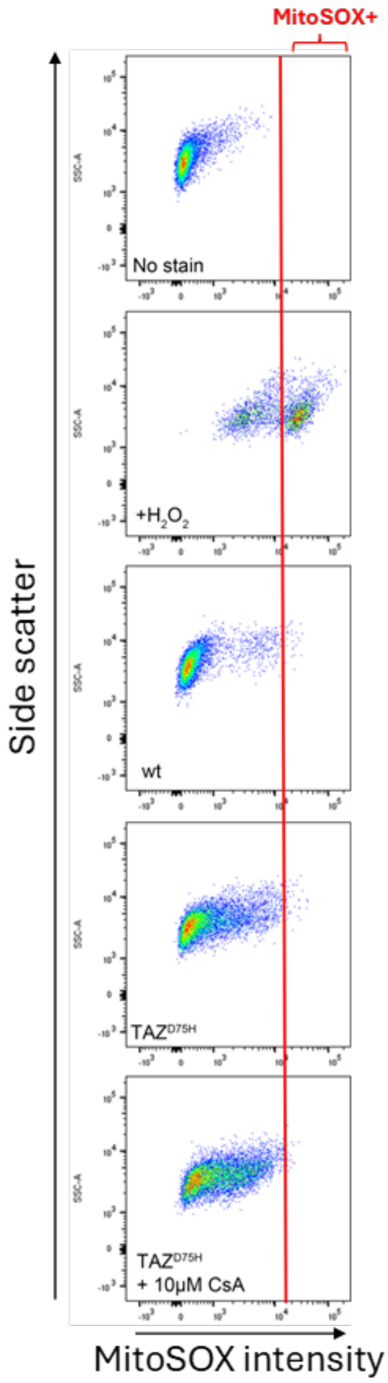

Supplemental Figure 8. Gating strategy to detect superoxide in lymphoblasts. Cells were stained with MitoSOX stain to detect superoxide via flow cytometry. Unstained cells were used as a negative control (top panel), and hydrogen peroxide-treated cells were used as a positive control to define the threshold for MitoSOX positivity (second panel). This threshold is marked by a red line through all panels. Representative plots of untreated wildtype, untreated TAZ^{D75H}, and CsA-treated TAZ^{D75H} lymphoblasts are shown in the bottom three panels.
